# Distinctive Membrane Accommodation Traits Underpinning the Neutralization Activity of HIV-1 Antibody against MPER

**DOI:** 10.1021/acs.molpharmaceut.4c01341

**Published:** 2025-04-09

**Authors:** Carmen Domene, Brian Wiley, Sara Insausti, Edurne Rujas, José L. Nieva

**Affiliations:** † Department of Chemistry, University of Bath, Claverton Down, Bath BA2 7AX, U.K.; ‡ Instituto Biofisika (CSIC, UPV/EHU), University of the Basque Country (UPV/EHU), P.O. Box 644, Bilbao 48080, Spain; § Department of Biochemistry and Molecular Biology, University of the Basque Country (UPV/EHU), P.O. Box 644, Bilbao 48080, Spain; ∥ Department of Pharmacy and Food Sciences, Faculty of Pharmacy, University of the Basque Country (UPV/EHU), Vitoria 01006, Spain; ⊥ Ikerbasque, Basque Foundation for Science, Bilbao 48013, Spain; ▽ ART-AI, Department of Computer ScienceUniversity of Bath, Claverton Down, Bath BA2 7PB, UK

**Keywords:** HIV-1 neutralization mechanism, MPER antibody, site-selective chemical modification, antibody engineering, antibody–membrane interaction, therapeutic antibody, MD simulations

## Abstract

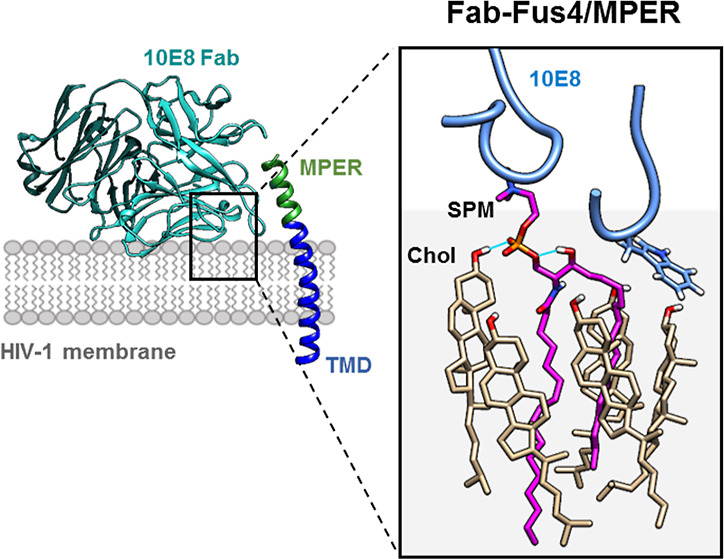

The membrane-proximal external region (MPER), located in the carboxy-terminal section of HIV’s envelope glycoprotein (Env) ectodomain, which is essential for viral entry into host cells, has gained considerable attention as a target for HIV vaccine development due to the exceptional neutralization breadth of antibodies against MPER epitopes. A distinctive feature of broadly neutralizing antibodies (bnAbs) targeting MPER is their requirement to accommodate the viral membrane into the surface of the antigen-binding fragment, or Fab moiety, to optimize antigen recognition. In this study, we sought to elucidate the molecular mechanism behind this interaction and its relevance to the antiviral function of bnAb 10E8. We conducted all-atom molecular dynamics simulations of three systems: (i) Fab 10E8 positioned on the surface of a viral-like lipid bilayer (VL-LB), (ii) Fab 10E8 in complex with an MPER helix anchored to the VL-LB via the Env glycoprotein transmembrane domain (TMD), and (iii) a Fab/MPER-TMD complex similarly embedded in the VL-LB but with a chemically optimized Fab 10E8 variant showing enhanced potency. Comparing these systems enabled us to derive atomic-scale Fab-membrane accommodation profiles pertinent to 10E8’s neutralizing function. Our findings support that Fab adaptation to the viral membrane interface following epitope binding is crucial for developing MPER-targeted neutralizing activity. This analysis also provides insights into pathways for strengthening lipid interactions, which may prove valuable in designing MPER-based biologics and vaccines to prevent or treat HIV infection.

## Introduction

The HIV/AIDS pandemic is still a major health problem worldwide, emphasizing the necessity of developing novel preventive and therapeutic approaches. In this endeavor, broadly neutralizing antibodies (bnAbs) that recognize the helical membrane-proximal external region (MPER) epitope at the base of the Env glycoprotein ectodomain constitute an important research focus due to their impressive neutralization breath, which can reach nearly pan-neutralizing levels in some instances.^1−6^ Vaccines targeting MPER have been recently evaluated in clinical trials,^7,8^ and scaffolds that stabilize MPER-based helices have been demonstrated to be capable of activating specific B-cell lineages.^9^

The ability for accommodating optimally the viral membrane appears to be crucial in the mechanism of neutralization of MPER bnAbs, even though the contribution of lipids may differ among the different classes.^10^ Particularly in the case of the bnAb class targeting the C-terminal subregion (ctMPER) joining MPER to the Env glycoprotein transmembrane domain (TMD), crystallographic studies revealed a common trend consisting of lipid-interacting and/or -accommodating pockets,^4,5,11−13^ which can sustain semi- or nonspecific interactions with the polar head-groups of membrane phospholipids.^14^

Thus, despite the fact that some ctMPER-targeting bnAbs do not display the capacity to bind membranes spontaneously (e.g., 10E8^2^), all of them share a surface adapted to accommodate phospholipid head groups during epitope recognition.^15^ Accordingly, mutations that favor interactions with the viral membrane lipids through this surface can increase the neutralization potency of ctMPER bnAbs, although moderately.^16−19^ In preceding work, we proposed an alternative pathway for improving the neutralizing capacity of these bnAbs based on the chemical modification with bulky aromatic compounds targeted to Fab-membrane contacts.^20,21^ We demonstrated that grafting this kind of compounds onto the framework region that must accommodate the viral membrane once the Ab-Env complex is formed^12,13,15^ enhances affinity and antiviral activity of 10E8 while preserving its breadth.^20^

Here, we have taken advantage of this potentiation mechanism and devised a computational approach to compare interactions with a virus-like lipid bilayer (VL-LB)^22^ of the apo form of the 10E8 Fab, its holo form bound to the ctMPER-TMD helix, and Fab-Fus4, a more potent, chemically modified 10E8 also in complex with ctMPER-TMD. By comparing conformational states and lipid interactions in these three systems, we were able to infer at the atomic-scale distinctive membrane-accommodation traits that correlated with the neutralization function of 10E8. Simulations of the Fab 10E8 apo form in contact with the VL-LB revealed spontaneous interactions with the constituent lipids, while changes in the lipid interaction network occurring after binding to ctMPER-TMD allowed us to distinguish those that are relevant for function. Finally, establishing the interactions that intensified after chemical modification of the Fab provided a more clear identification of function-related accommodation mechanisms and suggested unexplored pathways for improvement.

The results of these analyses provide valuable insights into the structural and functional dynamics of Fab 10E8 in relation to the viral membrane, which will inform the design of next-generation MPER-targeted biologics and vaccines. By pinpointing lipid interactions that are critical for antibody efficacy and identifying specific pathways for enhancing binding through chemical modification, our findings offer a foundation for optimizing antibody structure to improve potency, stability, and half-life. This knowledge has significant potential to advance HIV therapeutic strategies and accelerate the development of effective MPER-based vaccines aimed at broader and more durable immunity against HIV.

In a physiological setting, the 10E8 antibody functions within a complex immune environment, where interactions with immune cells, serum proteins, and host factors may influence its binding and neutralization mechanisms. While these aspects extend beyond the scope of our MD simulations, they represent important considerations for understanding 10E8 function in vivo. Additionally, compared to other MPER-targeting antibodies such as 4E10 and LN01, 10E8 exhibits distinctive membrane accommodation traits that contribute to its potent neutralization capacity. Its unique mode of interaction with the viral membrane, including specific lipid engagement and extended epitope recognition, likely plays a critical role in its efficacy. Future studies incorporating additional physiological factors will further refine our understanding of these mechanisms.

## Experimental Section

### Production and Site-Specific Chemical Modification of Abs

Methodologies described in ref (20) were applied to the production and chemical modification of 10E8 Fabs. In brief, to implement the site-directed procedure, we first generated a series of Fab 10E8 mutants bearing single, titratable Cys residues at defined positions, which were subsequently modified with a sulfhydryl-specific iodoacetamide derivative of the aromatic compound Fus-4. The shift of the MW detected by mass spectrometry after the procedure (262 Da) was consistent with the addition of a single Fus4 to the light chain (LC).

### Virus Production and Cell-Entry Assays

To produce JRCSF (Clade B, tier 2) pseudoviruses (PsV), human kidney HEK293-T cells were transfected with three plasmids as previously described,^20^ namely, pWXLP-GFP, which encodes a green fluorescent protein (GFP); pCMV8.91, encoding an HIV-1 genome lacking Env; and the full-length Env clone JRCSF (provided by Jamie K. Scott and Naveed Gulzar, Simon Fraser University, BC, Canada). Cell-entry inhibition activity of the 10E8 Fabs was subsequently measured using CD4+CXCR4+CCR5+ TZM-bl target cells (ARRRP, contributed by J. Kappes). Cells were grown in DMEM high glucose media +2 mM l-glutamine growth media, completed with 10% inactive FBS (fetal bovine serum, inactivated at 56 °C) and 50 μg/mL gentamicin, and incubated at 37 °C, 5% CO_2_. Serial dilutions (1:3) of the Fabs were incubated with 10–12% infecting dose of PsV for 1.5 h in 96 well plates. After incubation, 11.000 cells/well of TZM-bl cells were seeded in each well (supplemented with 25 μg/mL dextrans (Sigma-Aldrich, Steinheim, Germany)). After 72 h, the number of infected cells expressing GFP was determined by flow cytometry (CytoFLEX S, Beckman Coulter, IN, USA), and the IC_50_ value (i.e., the Fab concentration required for a 50% inhibition of the infection) was calculated by performing a nonlinear fitting of the experimental inhibition vs Fab concentration values using GraphPad Prism.

### Molecular Dynamics Simulations

#### System Setup

The starting model for the simulations was the X-ray crystal structure of the Fab-peptide complex (PDB ID 5GHW) at 2.40 Å resolution.^12^ The Fab-peptide complex is formed when the antigen-binding fragment (Fab) of broadly neutralizing antibody (bnAb) 10E8 binds to a peptide within the MPER of the HIV-1 gp41 protein, specifically the linear sequence of residues W672 to L683. This sequence is crucial for HIV’s membrane fusion process and is highly conserved across HIV strains, making it an ideal target for bnAbs like 10E8. Upon binding, 10E8’s Fab recognizes the peptide residues and interacts with the viral membrane, as the MPER epitope is near the lipid bilayer. This unique combination of peptide and lipid interactions stabilizes the Fab-peptide complex and enhances the antibody’s neutralizing potency. The Fab fragment of 10E8 includes the variable regions of the heavy and light chains, which form the binding sites for the MPER epitope. The epitope bound by 10E8 comprises a continuous helix spanning the gp41 MPER/TMD junction, including residues 671–687. Default protonation states were used for ionizable residues. The epitope was embedded palmitoyl-2-oleoyl-*sn*-glycero-3-phosphoethanolamine (POPE): *N*-acyl-sphingosine-1-phosphorylcholine (SPM): 1-palmitoyl-2-oleoyl-*sn*-glycero-3-phospho-*l*-serine (POPS): cholesterol (Chol), in a ratio 0.14:0.16:0.17:0.07:0.46, which mimics the viral membrane.^22^ The Fab-peptide orientation observed in the crystal structure was preserved. The bilayer was built using the CHARMM-GUI^24^ membrane builder module. The system was then solvated and neutralized using a 0.15 M KCl solution resulting in a simulation box of 170 × 170 × 160 Å^3^ dimensions. Following the experimental protocol, a chemical modification was first engineered to contain a single Cys residue at position 65, and subsequently, the Fus4, a synthetic aromatic compound, was used for antibody modification. Each system was composed of around 420,000 atoms.

Although real viral membranes exhibit a broader diversity of lipid species, dynamic fluctuations, and spatial heterogeneity, our chosen lipid composition (POPS/POPC/POPE/SPM/Chol) is based on experimentally validated studies that identify these as key components of the HIV envelope. This approach balances physiological relevance with computational feasibility, allowing us to capture essential biophysical properties of the viral membrane. While our model simplifies certain aspects of membrane complexity, it provides meaningful insights into the HIV membrane environment and its role in viral processes. Future work could extend this framework by incorporating additional lipid species and dynamic features to further refine the model.

The CHARMM36 force field was applied to model the Fab-peptide complex and lipids,^25^ using the TIP3P model for water^26^ and standard parameters for ions.^27^ The force field for the non-natural amino acid, consisting of an *S*-(2-amino-2-oxoethyl)-*l*-cysteine moiety with an attached Fus4 group, was developed by integrating existing parameters and generating any missing ones, followed by careful validation. Parameters for the *S*-(2-amino-2-oxoethyl)-*l*-cysteine moiety and the *S*-(2-amino-2-oxoethyl)-*l*-cysteine moiety with FUS4 had previously been established in refs (20 and 28).

The particle mesh Ewald method was used for the treatment of periodic electrostatic interactions, with an upper threshold of 1 Å for grid spacing.^29^ Electrostatic and van der Waals forces were calculated every time step. A cutoff distance of 12 Å was used for van der Waals forces. A switching distance of 10 Å was chosen to smoothly truncate the nonbonded interactions. Only atoms in a Verlet pair list with a cutoff distance of 16 Å (reassigned every 20 steps) were considered.^30^ The LINCS algorithm^31^ was used to constrain all bonds involving hydrogen atoms to allow the use of a 2-fs time step throughout the production phase.^32^ The multitime step algorithm Verlet-I/r-RESPA^33,34^ was used to integrate the equations of motion. The Nose–Hoover–Langevin piston method was employed to control the pressure with a 100 fs period, a 50 fs damping constant, and a desired value of 1 atm.^35,36^ Each system was coupled to a Langevin thermostat to sustain a temperature of 310 K throughout. The systems with the antibody were minimized using 10,000 steps and the steepest descent algorithm with an energy steep tolerance of 1000 kJ mol^–1^ nm^–1^. The equilibration consisted of six sequential steps in which the restraints of the protein backbone and side chain atoms, the lipid headgroups, and lipid torsions were progressively turned off. The equilibration protocol consisted of six steps, divided into two phases: an initial constant volume equilibration followed by a constant pressure equilibration. The constant volume equilibration steps were carried out for 125 ps using a 1-fs time step. During this phase, harmonic restraints were sequentially reduced on different parts of the system. The protein backbone atoms were restrained with force constants of 4000, 2000, and 1000 kJ·mol^−1^·nm^−2^. Restraints on the protein side chain atoms were reduced from 2000 to 1000 to 500 kJ·mol^−1^·nm^−2^, while lipid headgroup atoms were restrained with 1000, 400, and 400 kJ·mol^−1^·nm^−2^. Finally, lipid torsional restraints were progressively lowered to 1000, 400, and 200 kJ·mol^−1^·rad^−2^. The constant pressure equilibration steps were performed over 250 ps with a 2-fs time step, further reducing the harmonic restraints. Protein backbone restraints were gradually lowered from 500 to 200 to 100 kJ·mol^−1^·nm^−2^. Protein side chain restraints were reduced to 200 and 50 kJ·mol^−1^·nm^−2^, lipid headgroup restraints to 200 and 40 kJ·mol^−1^·nm^−2^, and lipid torsional restraints to 200 and 100 kJ·mol^−1^·rad^−2^. This stepwise relaxation ensured a smooth transition to an unrestrained state while maintaining system stability. Unconstrained dynamics was then performed for at least 1 μs for each system in triplicates. Simulations were performed with Gromacs 2020.4.^37^

Density profiles for each of the lipids and water at various vertical coordinates of the membrane were made with the VMD “Density Profile Tool” as an average over the three equilibrated systems and the first 100 ns of each of the replicas.^38^

Cluster analysis was done using the PyEMMA package.^39^ Trajectories were all concatenated together prior to running principal component analysis (PCA) for “Fab”, “Fab/MPER”, and “Fab-Fus4/MPER” systems, respectively. First, a displacement from the average of each atom position over the trajectory is calculated, and subsequently, a PCA is run on this displacement; the displacement from the total trajectory average was computed on a per Cα basis of the Fab, plus the 41-residue membrane-bound peptide in the case of the “Fab/MPER” and “Fab-Fus4/MPER” systems. Following extracting the first two primary principal components, the density was plotted using the “plot_density” function from PyEMMA with all the defaults. The density of the frame’s PCs plotted is not considered by k-means++ clustering, and thus, points were selected after visual inspection. From those clusters, frames were selected at the highest density of points within each cluster (red, yellow, and green in the figures).

### Statistical Analyses

Quantitative data were routinely displayed as means ± standard deviation (SD). The scipy.stats module (version 1.15.1) in python was employed to carry out the statistical analyses. Group comparisons were conducted using one-way analysis of variance (ANOVA) and pairwise Student’s *t* tests. Probability values *p* < 0.05 were considered reflecting statistically significant variances among samples.

## Results

### Viral Membrane-Accommodating Elements that Sustain Fab 10E8 Function

**Figure 1:**
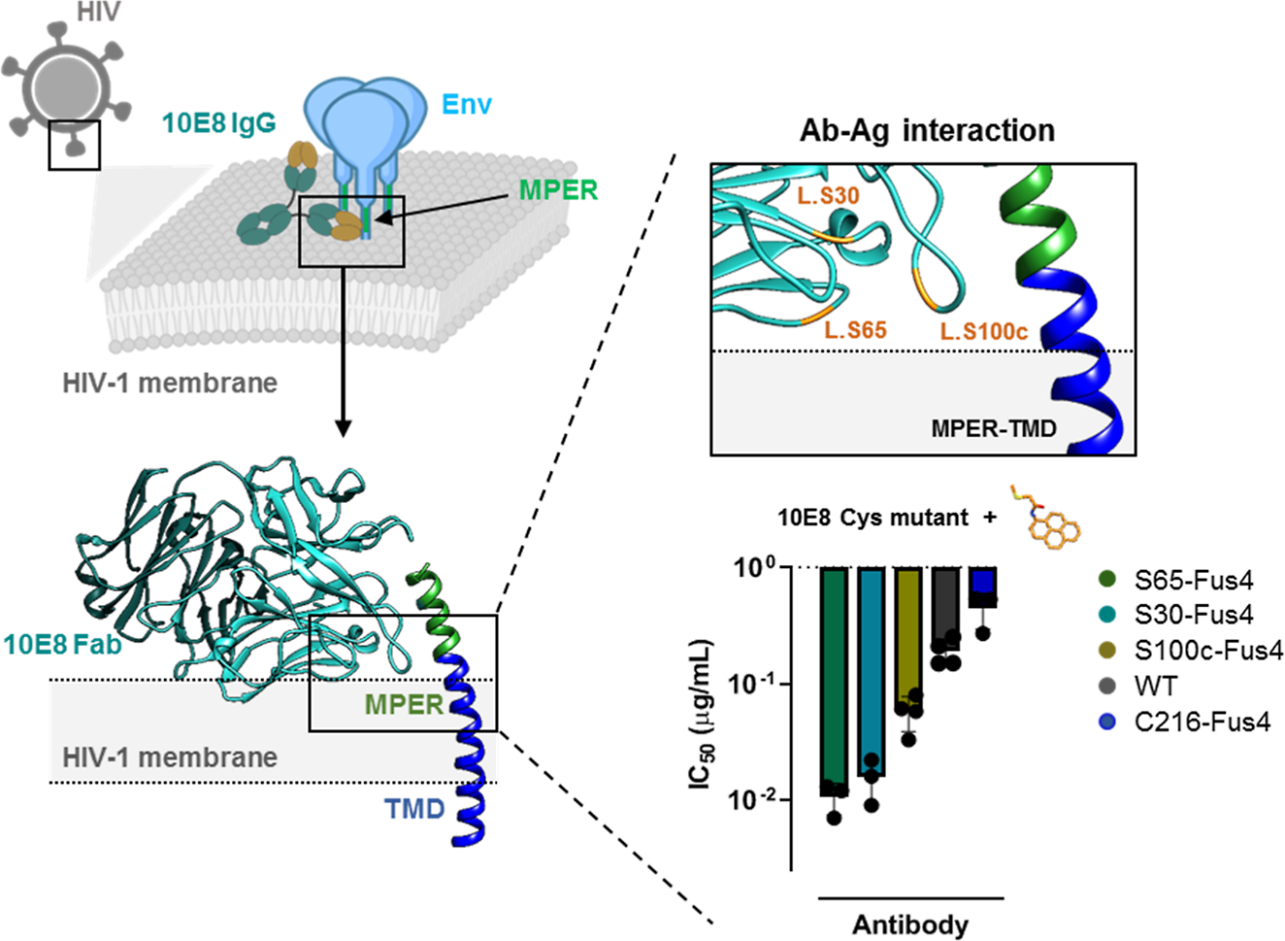
Probing the membrane-accommodating surface of Fab 10E8 through chemical modification with Fus4. Left: location within the HIV Env glycoprotein and the mode of recognition of the 10E8 MPER epitope. Right, top: solvent-exposed residues facing the membrane from the CDRL1 (LC.S30), FRL3 (LC.S65), and CDRH3 (HC.S100c), which were considered for single site-directed modification, are indicated in orange. Right, bottom: antiviral activity tested in cell entry assays. Reduction of the IC_50_ values (μg/mL) denoted higher neutralization potency of the antibody. A Fab modified after introducing a Cys at the *N*-terminus of the HC (C216-Fus4) was assayed as a negative control. Plotted values are means ± SD of three independent experiments (see also Supporting Information Figure S1A).

The location of the ctMPER helix and its recognition mode by 10E8 at the viral membrane interface based on cryo-EM structural data^15,40^ is depicted in the model shown in Figure 1 (left). The model shown on the right panel (top) further defines a Fab surface that accommodates the membrane, including loops derived from the complementarity-determining region 3 of the heavy chain (HC) (CDRH3), the complementarity-determining region 1 of the LC (CDRL1), and the framework region 3 of the LC (FRL3), all of which are implicated in direct binding to phospholipid headgroup moieties.^12,13^ To assess the functional relevance of these potential sites in membrane interaction, we first increased their interfacial hydrophobicity by grafting the bulky aromatic compound Fus4 onto the Fab surface at the following positions within these loops: the HC.Ser100c (CDRH3), LC.Ser30 (CDRL1), and LC.Ser65 (FRL3). Antiviral activities (cell-entry inhibition assays, right-bottom panel, see also Supporting Information Figure S1A) of the Fabs chemically modified at the selected positions were compared after the introduction of single, titratable Cys residues at those positions and the subsequent conjugation with the compound.^20^ Sustaining their predicted location at the viral membrane interface after engagement with the Env antigen, chemical modification of these sites enhanced the antiviral activity of the antibody. However, modification of FRL3 residue LC.Ser65 produced the Fab with the most pronounced activity improvement, whereas the modification that targeted CDRH3 residue HC.Ser100c increased activity to a lower extent. Thus, modification with Fus4 at a position distant from the ctMPER-binding pocket, hence, unlikely to a priori affect epitope specificity, rendered an optimized specimen of the 10E8 antibody. Consequently, we selected the Fab LC.Ser65Cys-Fus4 conjugate for subsequent computational analyses, as a potential tool to distinguish membrane accommodation traits related to the functional improvement of 10E8.

### Conformational Changes of the Fab Elements Accommodating the Viral Membrane from Atomistic MD Simulations

**Figure 2:**
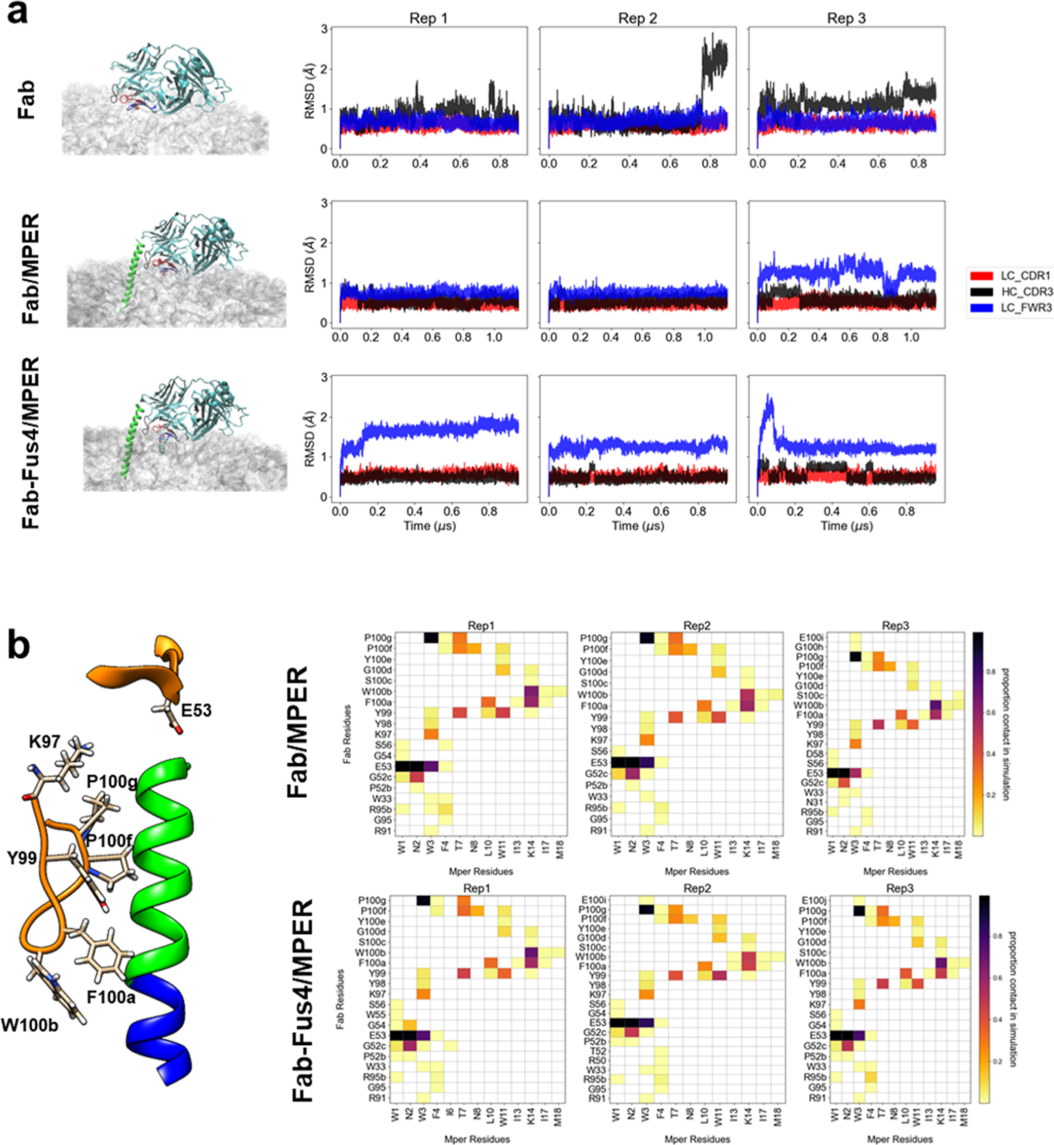
MD simulations of CDRL1, FRL3, and CDRH3 loops of the Fab 10E8 implanted into the VL-LB. (a) RMSD of three loop regions for each of the three systems. CDRL1 (LC Cys23-Ser34) in red, CDRH3 (HC Tyr98-Gly100h) in black, and FRL3 (LC Asp60-Ala78) in blue. The analysis was performed on frames extracted every 100 ps from each trajectory. (b) Specific Fab-MPER helix contacts in the “Fab/MPER” and “Fab-Fus4/MPER” systems were defined based on atomic proximity, where any heavy atom of an antibody residue (paratope/Fab) within 3.5 Å of any heavy atom in MPER (epitope) was considered a contact. The analysis was performed on frames extracted every 1 ns from each trajectory.

We performed simulations of three different systems (Figure 2a, left panels and Table 1). The first system comprised the apo form of the Fab deposited onto the membrane surface (designated as “Fab”; Figure 2a, left-top). The second system was assembled by implanting the Fab/ctMPER-TMD complex into the bilayer (designated as “Fab/MPER”; Figure 2a, left-center). For the assembly of this Fab/peptide complex, we utilized the X-ray crystal structure of 10E8 (PDB ID 5GHW)^12^ including the Fab bound to a continuous helix spanning the gp41 MPER-TMD junction, which was further completed by adding the TMD moiety from the crystal structure of the LN01/ctMPER-TMD complex (PDB ID 6SNE).^4^ The third system was obtained by replacing the Fab WT with a chemically modified Fab-Fus4 in the same complex (designated as “Fab-Fus4/MPER”; Figure 2a, left-bottom). To that end, the Ser65 of the FRL3 was first changed to Cys, and then the compound Fus4 was covalently attached through a modeled alkyl linker.^20^

**Table 1:** Average Root-Mean-Square Deviations (RMSDs, Å) ± SD of Fab Regions (CDRL1, FRL3, and CDRH) for the Fab, Fab/MPER, and Fab-Fus4/MPER Systems across Three Independent Replicas^a^

Fab region	system	RMSD (Å) replica 1	RMSD (Å) replica 2	RMSD (Å) replica 3
CDRL1	Fab	0.6 ± 0.1	0.6 ± 0.1	0.6 ± 0.1
	Fab/MPER	0.5 ± 0.1	0.5 ± 0.1	0.5 ± 0.1
	Fab-Fus4/MPER	0.6 ± 0.1	0.5 ± 0.1	0.5 ± 0.1
FRL3	Fab	0.6 ± 0.1	0.7 ± 0.1	0.7 ± 0.1
	Fab/MPER	0.7 ± 0.1	0.7 ± 0.1	1.2 ± 0.2
	Fab-Fus4/MPER	1.6 ± 0.2	1.2 ± 0.1	1.3 ± 0.2
CDRH	Fab	0.8 ± 0.2	0.9 ± 0.5	1.1 ± 0.2
	Fab/MPER	0.6 ± 0.1	0.5 ± 0.1	0.6 ± 0.1
	Fab-Fus4/MPER	0.5 ± 0.1	0.5 ± 0.1	0.6 ± 0.2

a The analysis was performed on frames extracted every 100 ps from each trajectory.

The VL-LB membrane model used in the three systems was based on a 5-lipid mixture of POPS/POPC/POPE/SPM/Chol, in a ratio 0.07:0.14:0.16:0.17:0.46, that was experimentally established to emulate the overall lipid composition and packing degree (rigidity) of the viral membrane.^22,41^ In the simulations of these three systems, we first analyzed the conformational changes undergone by the Fab 10E8 loops CDRH3, CDRL1, and FRL3 upon insertion into the VL-LB. The RMSD plots corresponding to those elements are shown in Figure 2a (right panels) in black, red, and blue, respectively. Regarding the overall stability, CDRL1 was identified as the most stable across all simulations (Table 1). In the “Fab” system, together with CDRL1, FRL3 was noted to be the most stable concerning their initial reference state, while the CDRH3 conformation appeared to be more variable during the simulations. In contrast, in the cases of the “Fab/MPER” and “Fab-Fus4/MPER” systems, the CDRH3 loop displayed the highest stability, and FRL3 showed a tendency to change its conformation with respect to the initial state (Table 1).

The comparatively lower stability of the CDRH3 loop in the apo form (“Fab” system) may indicate conformational changes associated with the unrestricted insertion and detachment of its hydrophobic tip at the membrane interface. In contrast, the conformation adopted by this element in the “Fab/MPER” and “Fab-Fus4/MPER” structures was constrained during the simulations due to specific interactions with the ctMPER helix epitope.^12^ Consistent with common structural behavior, the CDRH3 loop exhibited a similar set of contacts with residues of the ctMPER helix across both holo structures throughout the simulations (Figures 2b and S2a). Additionally, the angle between the main axis of the ctMPER helix and the membrane plane remained similar and stable across the simulations in both systems (Figure S2b). These data support our initial assumption that the enhanced antiviral function conferred by Fus4 conjugation did not result from a modified pattern of epitope recognition. Instead, the more pronounced conformational transitions observed in the FRL3 region, particularly in the most potent “Fab-Fus4/MPER” complex, suggest a correlation between changes in this region and a more efficient neutralization process.

### Lipid Interactions with Residues of Membrane-Accommodating Fab Elements

**Figure 3:**
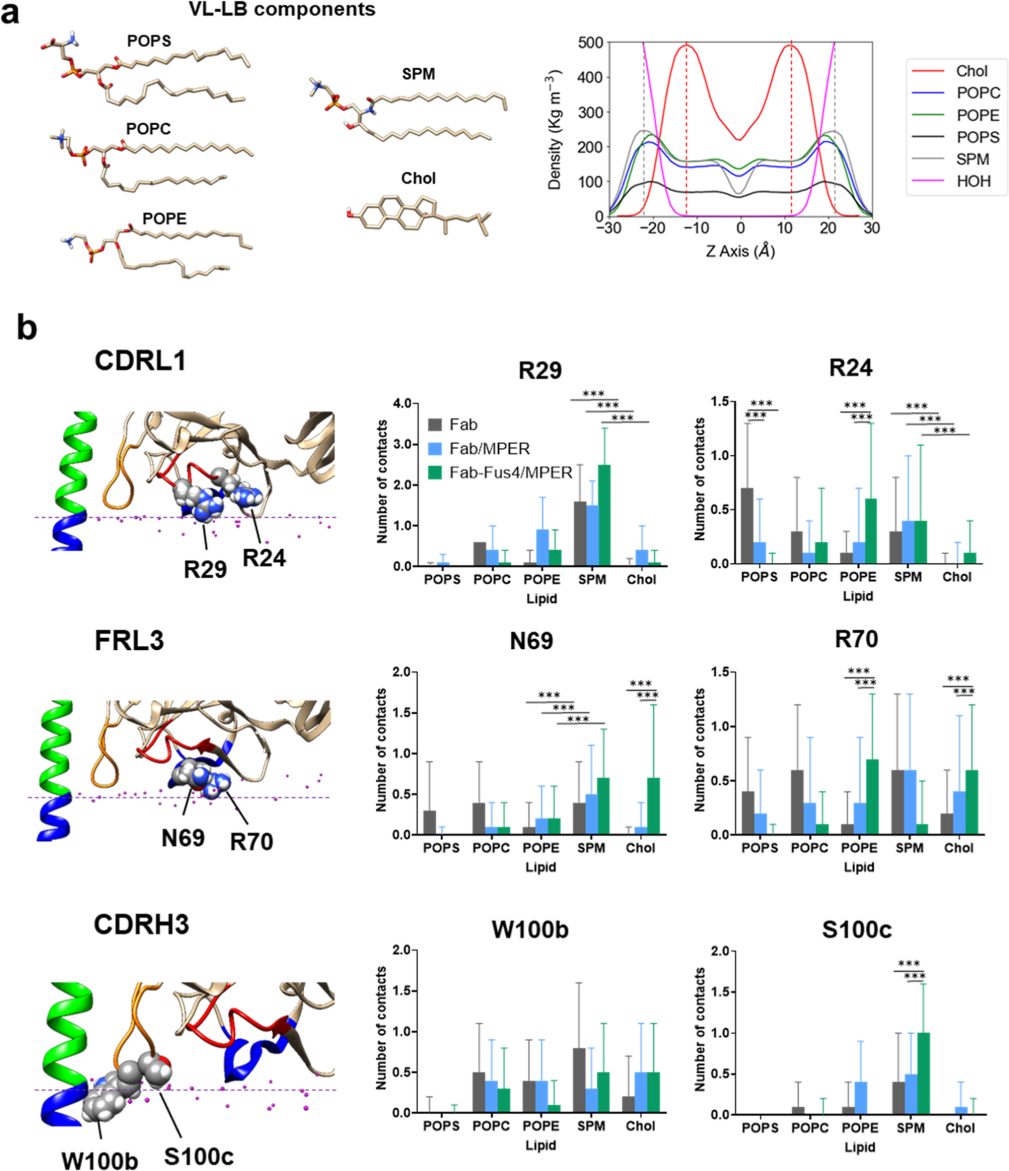
Interactions with VL-LB lipids of CRL1, FRL3, and CDRH3 loop residues in MD simulations of Fab 10E8. (a) Right: lipid components of the VL-LB mixture. Left: density profiles of the different VL-LB components. (b) Contacts with VL-LB lipids established by CDRL1, FRL3, and CDRH3 loop residues selected from maps displayed in Figure S2. Plotted values are means ± SD. Means were calculated after registering in frames collected at 1 ns intervals the number of contacts established by a given residue with a given class of lipid (heavy atom distance <3.5 Å) in Fab (gray), Fab/MPER (blue), and Fab-Fus4/MPER (green) simulations. *N* values amounted to 2662, 3480, and 2873 for Fab, Fab/MPER, and Fab-Fus4/MPER simulations, respectively. When contacts were detected, one-way ANOVA tests confirmed significant differences among the three simulations (*p* < 0.05). Horizontal bars indicate samples subjected to pairwise *t*-Student analysis (***p < 0.0005).

Next, to identify the residues responsible for most contacts with membrane lipids in the three systems, we generated contact maps for the Fab loops CDRL1, FRL3, and CDRH3 (Figure S3). From these maps, we selected two residues per loop that exhibited the highest contact density and examined their distinct interaction patterns with the different lipids in the VL-LB mixture (Figures 3 and S4). Two Arg residues within the CDRL1 loop were found to make most of the lipid contacts in this Fab element. On the one hand, LC.Arg24, located at a peripheral position of the paratope, exhibited a comparable interaction pattern across the three systems (Figure 3b, top). The most notable difference was the preferential interaction with POPS observed in the “Fab” system, which was replaced by interactions with POPE in the “Fab-Fus4/MPER” ensemble. On the other hand, LC.Arg29, a residue known to be involved in the configuration of the 10E8 phospholipid-binding site,^13^ preferentially interacted with SPM in all three systems, with a stronger interaction in “Fab-Fus4/MPER”.

None of these two CDRL1 residues appeared to interact effectively with Chol during the simulations (Figure S4), despite Chol being present in a higher proportion in the VL-LB mixture compared to individual phospholipids. The mass density profiles of the VL-LB lipids revealed that Chol was located deeper within the bilayer (Figure 3a, right). Therefore, the absence of interactions between LC.Arg24 and LC.Arg29 with Chol suggests that the side chains of these residues subtly insert into the membrane interface, with lipid contacts confined to the bilayer section occupied by phospholipid headgroup moieties.

In contrast, the two residues identified within the FRL3 loop, LC.Asn69 and LC.Arg70, exhibited a significant number of contacts with Chol in the “Fab/MPER” and “Fab-Fus4/MPER” systems, a phenomenon that was more evident in the latter one, representing the most potent 10E8 activity. In fact, interactions of LC.Asn69 in this system were mainly restricted to SPM and Chol. In comparison, in the “Fab” system, LC.Asn69 established interactions with all phospholipids, including POPS, but not with Chol (see also Figure S4). More distal LC.Arg70 interacted preferentially with POPE and Chol in the “Fab-Fus4/MPER” system, whereas the opposite pattern was observed in the “Fab” simulations, i.e., LC.Arg70 mainly interacted with POPS, POPC, and SPM, but barely with POPE or Chol, in that system.

Thus, the interaction traits of the FRL3 residues were overall consistent with the conformational change and deeper membrane insertion of this Fab loop in the “Fab-Fus4/MPER” system, which may facilitate contacts with Chol molecules (see also the Clusters Derived from the Analysis of Principal Components below). In contrast, in the case of the free “Fab” system, more subtle contacts with phospholipid head-groups seemed to underlie its mechanism of membrane accommodation.

Finally, we compared interactions of two CDRH3 residues, HC.Trp100b and HC.Ser100c, which were previously described to insert into the membrane^12^ and to contribute to phospholipid binding,^13^ respectively (Figure 3b, bottom). In the case of the HC.Trp100b, the interaction patterns seemed similar for the three systems, showing contacts with Chol and certain preference for SPM over glycerophospholipids (see also Figure S4). In contrast, HC.Ser100c displayed a clear preference for SPM, the highest number of contacts with this lipid scored in the “Fab-Fus4/MPER” simulations.

### Accommodation of the Phospholipid Binding Site

**Figure 4:**
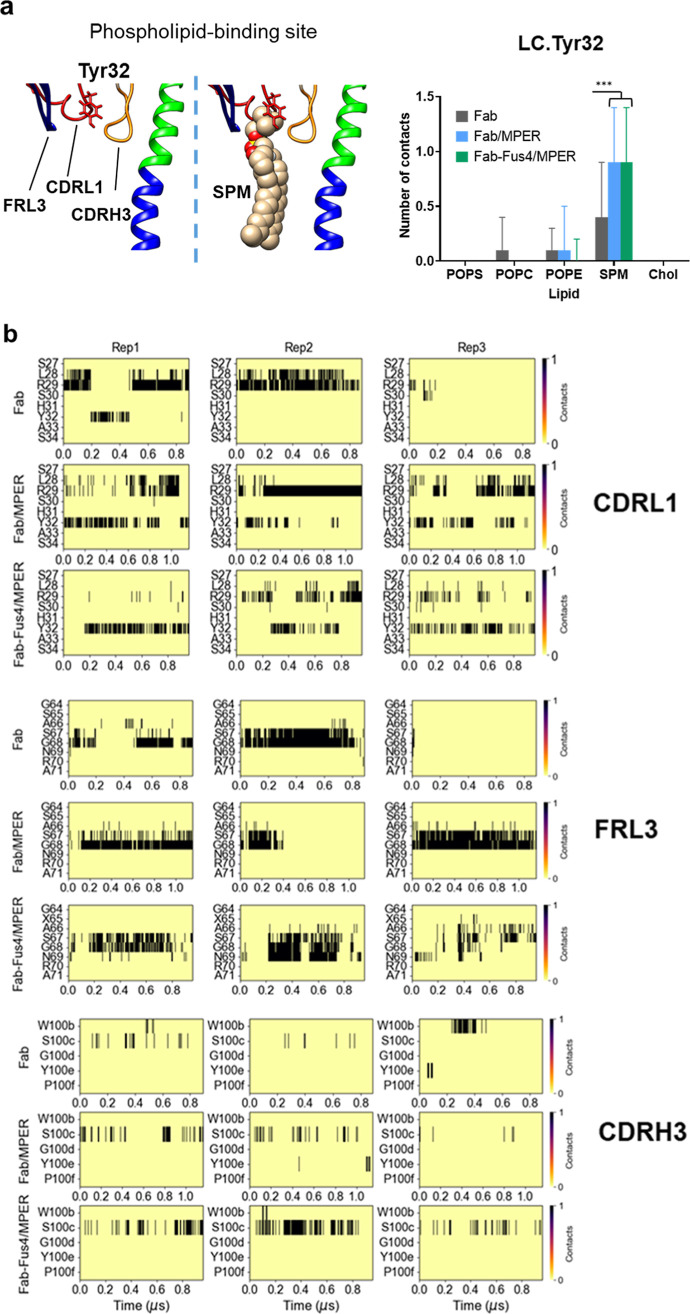

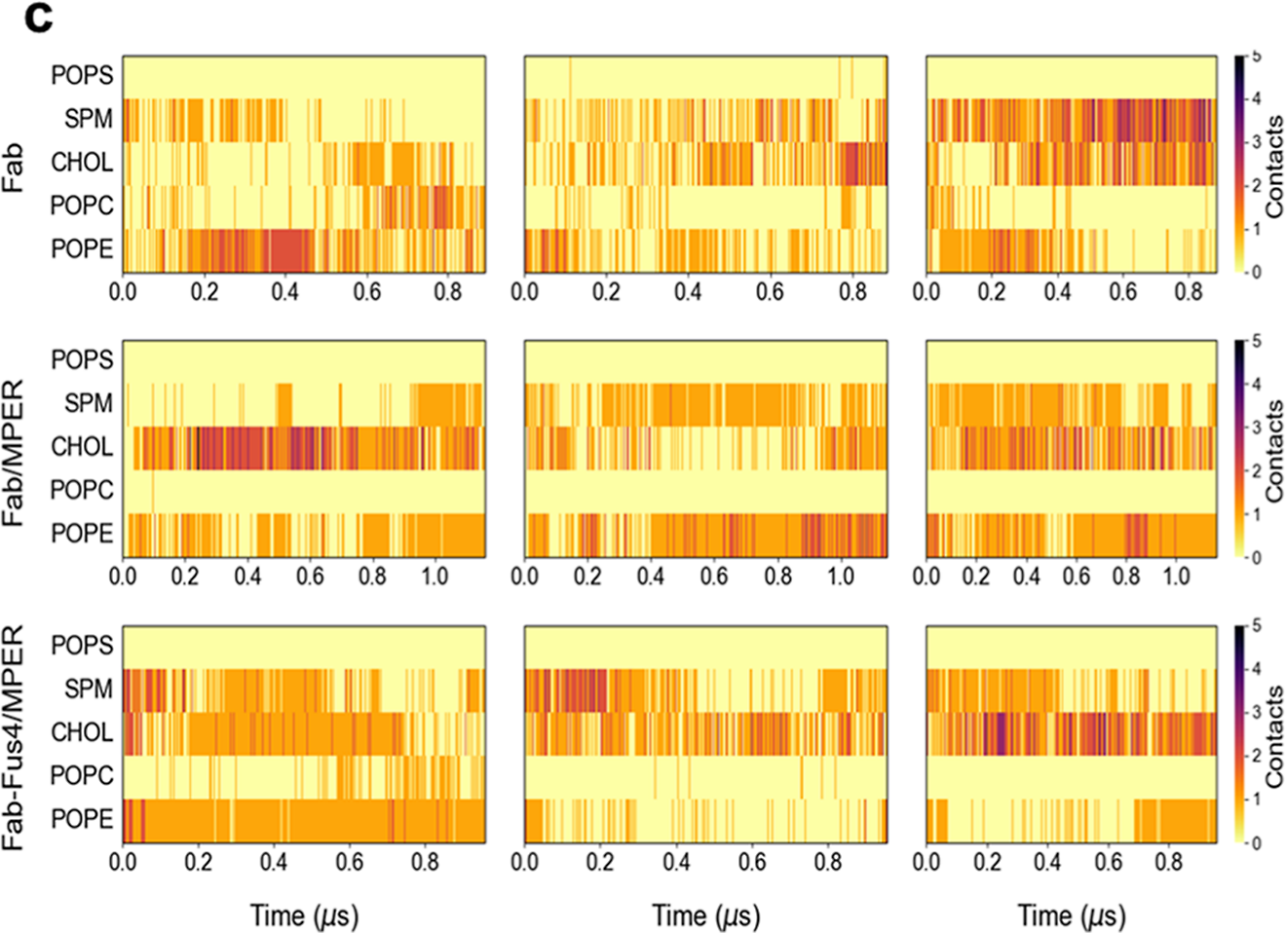
SPM filling of the binding site in the MD simulations of Fab 10E8. (a) Structure of the binding site (left) and number of contacts (heavy atom distance <3.5 Å) established by the LC.Tyr32 residue with the different VL-LB lipids (right). Conditions otherwise as in previous Figure 3b. (b) Contacts (heavy atom distance <3.5 Å) of SPM phosphocholine moiety with Fab residues from CDRL1, FRL3, and CDRH3 loops in simulations of the three systems. Black represents the maximum of 1 contact between single residue and phosphocholine headgroup. (c) Contacts established with VL-LB lipids of the same monolayer by the SPM molecule that remained in contact with Tyr32 in most of the trajectories. Number of contacts measured using frames at 1 ns intervals, between the SPM residue, with its headgroup occupying the cavity formed around the residue Tyr32 of the LC-CDR1 loop, and the other lipids in the upper leaflet of the membrane.

The CDRL1-Tyr32 residue occupies the roof of the phospholipid-binding site, positioned the furthest from the bilayer plane (Figure 4a, left). To explore 10E8 binding specificity, we quantified lipid contacts with LC.Tyr32 as a way to monitor highly specific Fab interactions with lipid components of the VL-LB mixture (Figures 4a, right and S4). The data confirmed that SPM preferentially occupied this site in the “Fab” system, while the site was almost exclusively occupied by SPM in the “Fab/MPER” and “Fab-Fus4/MPER” systems. In some simulations, the SPM was found to remain within 3.5 Å of LC.Tyr32 throughout the entire trajectory. In other simulations, POPC/E molecules occasionally came within 3.5 Å, but these interactions were more sporadic compared with those with SPM.

The SPM phosphocholine moiety had to protrude from the bilayer plane to insert into the Fab cavity formed by the CDRL1, FRL3, and CDRH3 residues (Figure 4a, left). Given the distinctive conformational changes observed for the FRL3 and CDRH3 loops in the three systems (Figure 2a), we next questioned whether the set of contacts established by the SPM phosphocholine moiety with Fab residues might vary across the systems. As shown in Figure 4b, moving from the lowest (“Fab”) to the highest (“Fab-Fus4/MPER”) functional competence, the frequency of contacts with LC.Tyr32 of CDRL1 increased significantly. For FRL3, the Ser67/Gly68 residues positioned at the loop β-turn established most of the contacts in the three systems. However, consistent with the observed conformational changes in this loop, there was a clear tendency for an increase in the number of residues contacting the bound phosphocholine in the “Fab-Fus4/MPER” system. Most notably, LC.Asn69 established a significant set of contacts, which were absent in the other systems, while the number of contacts with LC.Ala66 increased significantly. These observations indicate a reconfiguration of the SPM-binding site that is linked to functional improvement. Changes in the phosphocholine interaction pattern were also observed in the case of the CDRH3 loop. In this loop, the function-related signature appeared to be the establishment of short-distance phosphocholine-HC.Ser100c interactions.

Given the capacity of the Fab 10E8 for binding different types of phospholipids,^12,13^ it was also unclear why, among the phospholipids present in the VL-LB mixture, SPM was selected as the preferential ligand. The raft-like composition of the viral membrane endows it with the ability to assemble laterally segregated nanodomains.^22^ SPM is known to form intermolecular hydrogen bonds between molecules and with colipids, and it also packs more favorably with certain bilayer components when codispersed in raft-like mixtures.^42−44^ Notably, among the lipids present in the VL-LB, SPM can potentially establish strong interactions with Chol through favorable packing constraints and/or interfacial hydrogen bonding involving its amine group and the hydroxyl group of Chol.^42−44^ Thus, we next investigated whether a particular nanoenvironment surrounding the bound SPM molecule could sustain Fab recognition of its phosphocholine moiety.

The maps reporting intermolecular contacts of the SPM molecule, acting as a ligand, with other lipids in the same monolayer confirmed interactions with additional SPM molecules (Figure 4c). This set of contacts, along with those established with POPE, accounted for most contacts in the “Fab” system. In the trajectories of the “Fab/MPER” and “Fab-Fus4/MPER” systems, Fab-bound SPM molecules displayed a higher number of contacts with Chol, consistent with its known affinity. In contrast, contacts with POPC/S were less frequent in the “Fab” system and absent in most trajectories of the “Fab/MPER” and “Fab-Fus4/MPER” systems. Thus, the SPM ligand showed a tendency to cluster with Chol and POPE in the systems that represent functional interactions with the VL-LB membrane. Interestingly, although SPM-Chol mixtures have a characteristic tendency to segregate laterally into microdomains, some studies suggest that monounsaturated POPE may remain comixed with these compounds.^45^ Based on this evidence, we speculate that the large phosphocholine headgroup may protrude from the lipid bilayer and become more accessible when the SPM ligand is surrounded by POPE and Chol, the two VL-LB lipids with the smallest polar head groups that interact favorably with SPM (see below).

### Clusters Derived from the Analysis of Principal Components

**Figure 5:**
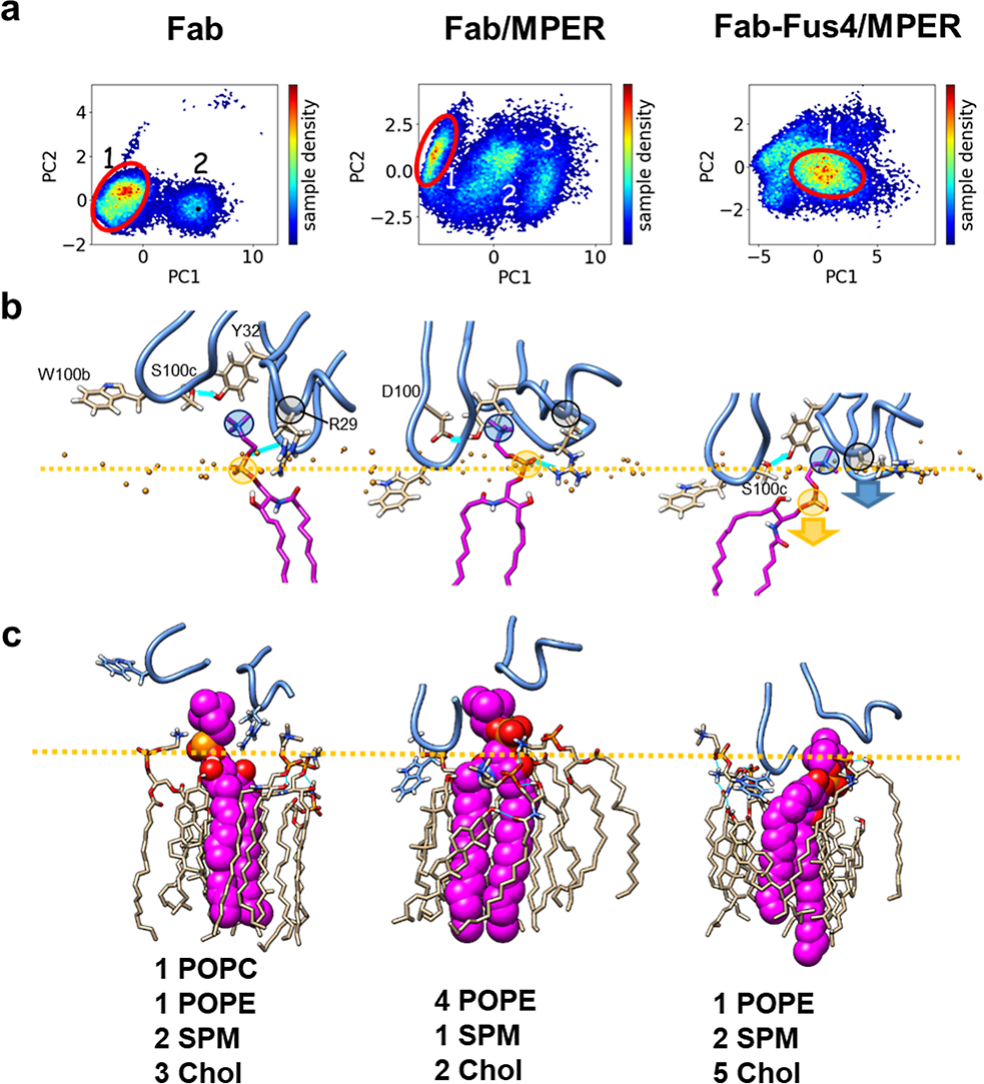
Phospholipid-binding sites in structures derived from cluster analyses. (a) Cluster analysis of “Fab”, “Fab/MPER”, and “Fab-Fus4/MPER”. (b) Position of the phospholipid-binding site with respect to the bilayer plane in the extracted structures (membrane phosphate level indicated by the orange line). Side-chains of HC.W100b, HC.S100c, LC.Tyr32, and LC.Arg29 are displayed for better appreciation of the displacement of the constituent loops. The bound SPM molecule depicted in magenta portraits choline and phosphate groups encircled (blue and orange circles, respectively). Position of the LC.Arg29 Cα is indicated by the gray circle. Intramolecular (HC.Ser100c-LC.Tyr32 and HC.Asp100-LC.Tyr32) and intermolecular (SPM-LC.Gly68 and SPM-LC.Arg29) H-bonds are highlighted by the lines in cyan. (c) Lipid nanoenvironment surrounding the SPM ligand. VL-LB lipids making contact at a distance <3.5 Å are depicted together with the Fab-bound SPM molecule (VW surface in magenta).

PyEMMA cluster analysis provided additional evidence confirming the distinctive conformational states and interactions present during the MD simulations of the three systems.^39^ For cross-validation and model selection to ensure the robustness and reliability of the results, we used different tools available in PyEMMA and inferred different numbers of clusters to conclude with the results presented in Figure 5a. The frames that were near the highest cluster densities (Figure 5a in red) were manually chosen from the density plots, and the corresponding structures were extracted for further 3D analysis.

Figures 5b and S5 compare the positions of the CDRL1, FRL3, and CDRH3 loops and bound SPM molecules with respect to the bilayer plane in structures derived from the densest clusters. In the absence of the bilayer-anchored MPER epitope (“Fab” system, Figure 5b, left panel), the phospholipid-binding site of the Fab presented an open configuration on one of its flanks when compared to the Fab structure obtained for the system with the ctMPER-TMD peptide bound (“Fab/MPER” system, center panel). In both structures, the top of the cavity and one of its flanks were occupied by CDRL1 residues LC.Tyr32 and LC.Arg29, respectively, with the side chain of the latter projecting almost perpendicular to the bilayer plane. The main difference pertained to the CDRH3 position on the opposite flank, whose tip inserted more deeply into the interface in the “Fab/MPER” structure (see also Figure S5).

**Figure 6:**
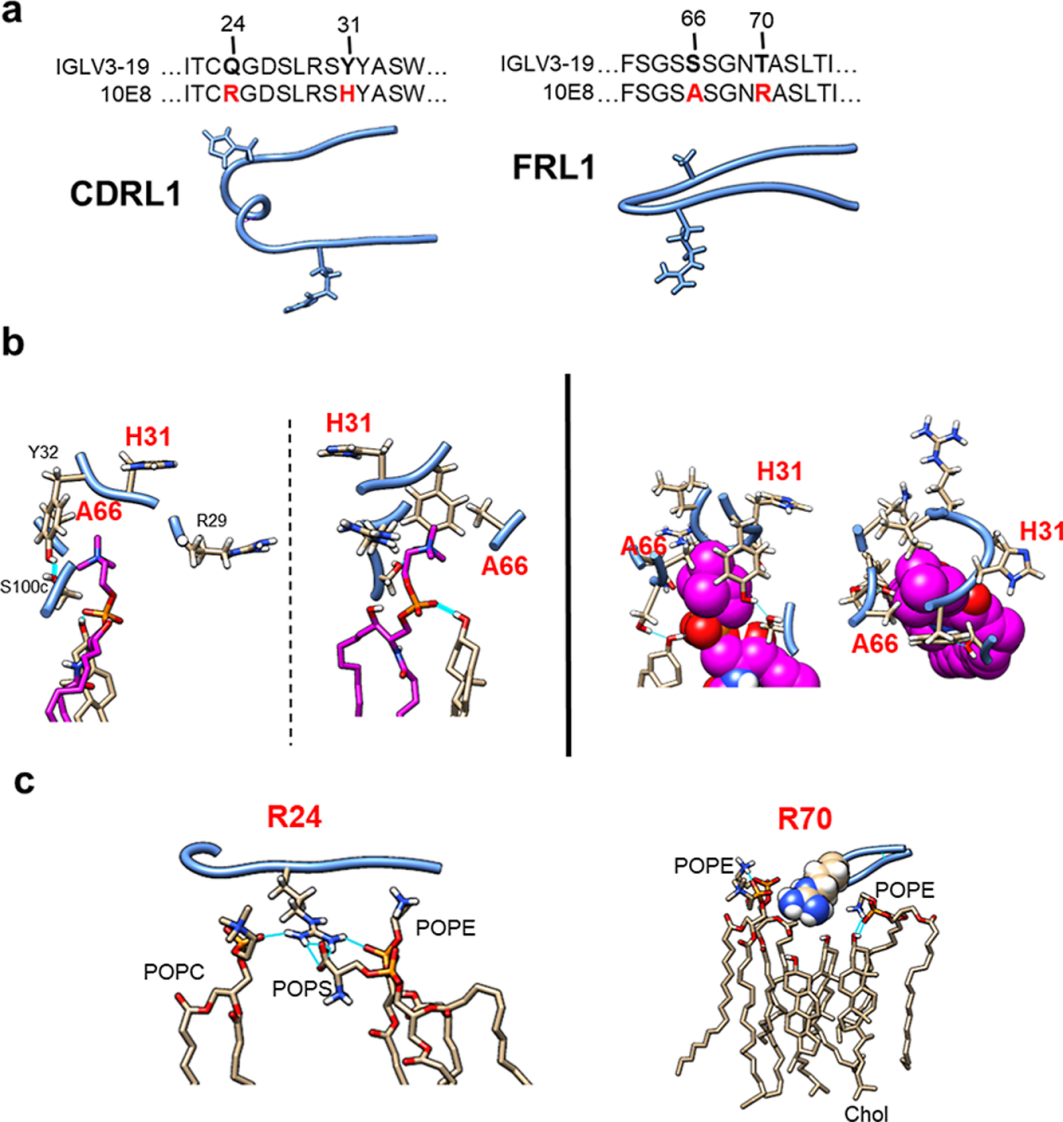
Somatically mutated residues in membrane-accommodating surfaces of the functional Fab 10E8. (a) Positions in the sequence and structures of the CDRL1 and FRL3 loops of somatically mutated residues (bold letters in red). IGLV3-19 sequence based on allele 01 (UNIPROT KB accession number: P01714). (b) Models depicting the position of LC.His31/LC.Ala66 in the SPM-binding site of the “Fab-Fus4/MPER” structure derived from the cluster analysis. Side chains of displayed residues established contacts with the phosphocholine moiety at a distance <3.5 Å. (c) Interactions with VL-LB lipids (distance <3.5 Å) of LC.Arg24 and LC.Arg70 in the same structure.

Further underscoring a different mode of SPM ligand binding, LC.Tyr32 capped the cavity by forming distinct hydrogen bonds with CDRH3 residues HC.Ser100c and HC.Asp100 in the “Fab” and “Fab/MPER” structures, respectively (Figure S4). In line with these changes, the bound SPM molecule also interacted differently with Fab and VL-LB lipids in these two systems (see Figure 6 below). In the “Fab” system, the FRL3 Gly68 amide established an H-bond interaction with the SPM phosphate, whereas in the “Fab/MPER” system, this ligand group was H-bonded to the guanidine of LC.Arg29.

In contrast to these two structures, in the “Fab-Fus4/MPER” structure (Figure 5b, right panel), all elements of the phospholipid-binding site, including the bound SPM ligand, inserted deeper into the membrane interface. Deeper insertion of the CDRL1 and FRL3 loops in this system was accompanied by the recapitulation of H-bonding between CDRH3 HC.Ser100c and CDRL1 LC.Tyr32, the conformational change of the LC.Arg29 polar side chain (which now lay flat on the membrane surface), and a change in the SPM headgroup conformation that allowed penetration of its phosphate group to the level of the sphingosine backbone (Figure 5b, right). Moreover, the FRL3 loop underwent a conformational change upon insertion, consistent with the elevated RMSD of this element in the Fab-Fus4/MPER system (Figure 2).

We surmised that these changes in the penetration level of the Fab elements might also impact the lipid nanoenvironment surrounding the SPM ligand. Panels in Figure 5c compare the lipids in contact with the SPM ligand molecule in the three structures derived from the high-density clusters (see also Figure S6a). In all three structures, POPE and Chol seemed to facilitate the access of the SPM phosphocholine moiety to the binding site, as well as help accommodate the membrane onto the Fab’s contact surface as previously noted (see Figure 4c).

Additionally, a set of SPM-*co*-lipid interaction motifs appeared to contribute to phosphocholine binding and the efficient accommodation of the membrane onto the Fab backbone (Figure S6b). Saturated acyl chains of neighboring SPM molecules were observed in contact with the Fab-bound SPM molecule, while intramolecular hydrogen bonding between the hydroxyl group and the oxygen atom of the phosphoether seemed to provoke the folding of the phosphocholine moiety, acquiring a de facto conical shape and reducing its surface area (Figure S6b, left). Intra- and intermolecular hydrogen bonding between the primary amine and the phosphate or carboxyl groups of POPE seemed to exert a similar effect (Figure S6b, center).^46^ In this context, the deeper insertion of the complex in the most functionally competent “Fab-Fus4/MPER” structure appeared to be aided by a higher accumulation of Chol molecules underneath the SPM ligand and the Fab elements associated with it (Figure S6b, right).

Given the suggested importance of the 10E8 maturation process for the generation of a membrane-accommodating Fab surface, we finally examined the roles of somatically mutated residues LC.Arg24/LC.His31 and LC.Ala66/LC.Arg70, which were present in the CDRL1 and FRL3 loops, respectively, in the configuration of the phospholipid-binding site and the establishment of Fab-lipid interactions (Figure 6a).

On the one hand, we found that two of these residues, CDRL1 LC.His31 and FRL3 LC.Ala66, became significant for the configuration of the phospholipid-binding site in the structure representing the “Fab-Fus4/MPER” system (Figures 6b and S7a). The LC.His31 contacts appeared to be mediated through the Cα backbone, suggesting that this residue stabilizes the CDRL1 loop in a conformation favorable for interaction. Along with those from LC.Leu28/LC.Tyr32, the side chain of LC.Ala66 projected toward the cavity containing the SPM phosphocholine, contributing a nonpolar surface. This binding-site reconfiguration was exclusively observed in the “Fab-Fus4/MPER” structure. Thus, interactions with the ligand established by the somatically mutated LC.His31 and LC.Ala66 residues, combined with the overall reconfiguration of the binding site in the structure representing the highest density “Fab-Fus4/MPER” cluster, support the involvement of an SPM-binding site deeply inserted into the viral membrane in efficient 10E8-mediated neutralization.

On the other hand, LC.Arg24 and LC.Arg70 established distinctive interactions with VL-LB lipids (Figures 6c and S7b). The guanidine group of CDRL1 LC.Arg24 formed H-bond interactions with different phospholipid groups in all three systems, including the phosphates of SPM, POPC, and POPE and the carboxyl group of POPS. However, reflecting a distinct insertion pattern, the number of interactions was higher in the “Fab-Fus4/MPER” structure. The differences between systems were more pronounced in the case of FRL3 LC.Arg70. Interactions of this residue with lipids at distances <3.5 Å were absent in the structure representing the “Fab” cluster but frequent in the “Fab/MPER” and “Fab-Fus4/MPER” structures. Consistent with changes in the FRL3 loop conformation in this latter system, deeper insertion of LC.Arg70 appeared to be supported by the accumulation of Chol molecules beneath the Cα backbone and its side chain.

## Discussion

The full antigenic structure recognized by the bnAb 10E8 comprises, in addition to the conserved ctMPER epitope sequence, glycan and lipids, derived from the Env glycoprotein and viral membrane, respectively.^15,40^ The overarching architecture of this complex antigen model implies that generation of a Fab surface that optimizes interactions with the viral membrane (i.e., lipid polyspecificity) is key for developing 10E8 neutralization activity during maturation.^4,15,16,47−49^ Supporting the functional role of ancillary interactions with envelope lipids, strengthening of 10E8 Fab-membrane interactions through mutagenesis,^16,17,19^ or more recently, through targeted chemical modification,^20^ can enhance binding to Env antigen and viral neutralization. The strategy of increasing membrane lipid affinity for developing more potent MPER-targeting bnAbs may be comparatively advantageous because of the invariant nature of the lipid envelope, which may prevent the potential emergence of viral resistance to Ab action. In addition, compared with traditional strategies based on amino acid residue substitutions, chemical conjugation with synthetic compounds may enable Ab modifications within a wider range of the physicochemical space. Together, these conditioning factors open up new prospects for the development of 10E8-based biologics with an optimized therapeutic profile. However, the set of Fab-membrane interactions that initially sustained 10E8 antiviral activity and subsequently potentiated it upon chemical modification remained undefined.

Here, by running MD simulations of the “Fab”, “Fab/MPER”, and “Fab-Fus4/MPER” systems, we were able to get atomic-scale insights into the 10E8 interaction with virus-like membranes under functional conditions. The comparison of these three systems revealed that the CDRL1 conformation was the most stable element across all simulations, while CDRH3 and FRL3 exhibited varying degrees of stability, depending on the simulation conditions. Specifically, CDRH3 appeared to undergo conformational changes mainly circumscribed to the “Fab” system that represented the apo form of the antibody, whereas FRL3 showed the most significant departure from the initial state in simulations of the “Fab-Fus4/MPER” system, which represented the condition of highest neutralization competence. Importantly, conformation of the CDRH3 appeared to become fixed through interactions with the ctMPER helix in the holo forms “Fab/MPER” and “Fab-Fus4/MPER” systems. Thus, when taking the apo form of the Fab and its spontaneous interactions with the VL-LB as the reference state, the analysis pinpointed the CDRH3 and FRL3 loops as the Fab elements in contact with membranes that underwent conformational changes related to function. The observation that HC.Trp100b and LC.Asn69/LC.Arg70 residues within these elements could establish contacts with Chol further supported their deeper insertion into the membrane interface.

Further analysis revealed structural differences in the Fab loops interacting with the membrane across the three systems; we identified distinct clusters sampled during the MD simulations, highlighting structural heterogeneity and multiple stable states. Comparisons of the most populated cluster structures revealed conformational differences among the systems. In the representative structure of the “Fab” cluster 1, the CDRH3 tip remained in solution, suggesting that conformational changes in this loop during the trajectories could allow transitions from its initial membrane-inserted state. In contrast, the FRL3 loop showed the greatest difference in the representative structure of the single cluster detected for the “Fab-Fus4/MPER” system, where it adopted a conformation that penetrated more deeply into the membrane interface. This deeper insertion appears functionally relevant, as LC.Arg70, a residue added to FRL3 during maturation, formed an increased number of contacts with viral membrane lipids.

Another structural feature distinctively associated with function was the configuration and position with respect to the membrane plane of the phospholipid-binding site and its bound ligand. Previous experimental work demonstrated that a double LC.Arg29Ala/LC.Tyr32Ala substitution resulted in a 10E8 variant capable of binding an analogue of the ctMPER helix but devoid of antiviral activity.^13^ Thus, the observation that CDRL1 residues LC.Arg29 and LC.Tyr32 established interactions almost exclusively with SPM in the system representing the highest neutralizing activity was consistent with the role of specific binding to this lipid for efficient 10E8-mediated neutralization. Notably, the set of residues from the FRL3 and CDRH3 loops in direct contact with the Fab-bound SPM phosphocholine moiety changed in the simulations, supporting a reconfiguration of the phospholipid-binding site in the systems representing the functional state of the Fab.

Cluster analyses further emphasized the differences existing in the SPM-binding sites of the three systems, the most notorious ones being reflected by the distinct configurations adopted in structures with the bound ctMPER epitope and their positions with respect to the bilayer plane. In the “Fab-Fus4/MPER” structure, the phospholipid-binding site appeared to submerge into the membrane interface by ca. 4–5 Å, an effect sustained by gathering Chol molecules around the SPM ligand and underneath the Fab elements implicated in its binding. Moreover, the somatically mutated LC.Ala66 residue established contacts at short distance with the SPM ligand bound to the phospholipid-binding site, in consonance with a reconfiguration of the phospholipid-binding site in going from the apo form represented by the “Fab” system to the most functionally competent form represented by the “Fab-Fus4/MPER” system.

Collectively, these factors—enhanced hydrogen bonding, membrane ordering effects, and potential cholesterol priming—boost the exposure of SPM’s phosphocholine group (over that of PC) by promoting a particular lipid nanoenvironment enriched in Chol, which also facilitates membrane integration of the Fab structural components. Thus, in this lipid context, SPM’s phosphocholine moiety protrudes from the bilayer plane, while CDRL1 and FRL3 loops may insert deeper. These observations are further supported by our contact analysis based on Fab residues (LC.Arg29, LC.Tyr32, HC.Ser100c, and HC.Trp100b interactions) and lipids surrounding the SPM ligand.

Our data also provide some clues on the mechanism sustaining the binding specificity observed during the simulations. Even though SPM and POPC were included at similar mole ratios in the VL-LB composition, and both shared the same phosphorylcholine moiety as polar headgroup, the former was exclusively found to occupy the binding site in the functionally meaningful systems. Thus, the structural basis underlying the binding selectivity of the former over the latter was not obvious. Specific bonding of sphingomyelin through its amine group, hydroxyl group, and carbonyl oxygen is essential for binding affinity and catalytic activity of sphingomyelinase enzymes.^43^ One possible explanation for this preference is that specific groups in SPM, which are absent in POPC, might act as potential hydrogen donors or acceptors, thereby conferring interaction specificity.^42,51^ Although simulations and cluster structures did reveal intermolecular hydrogen bonds between the SPM phosphate group and Fab residues, no interactions were detected through the donor–acceptor groups unique to SPM, ruling out this possibility. Instead, we explored the alternative that SPM becomes a more accessible ligand through specific interactions with other components of the VL-LB mixture. SPM molecules can form intermolecular hydrogen bonds with cholesterol and tend to pack more favorably with it when codispersed in mixtures.^42−44^ Analyses of the trajectories and clusters revealed that the phosphocholine of SPM could project outward when surrounded by POPE and Chol both possessing small head groups. Therefore, we surmise that the lipid nanoenvironment provided by the raft-like composition of the viral membrane may play an important role in phospholipid binding affinity and membrane accommodation of 10E8.

As a final consideration, we envision future analyses that could confirm and complement our observations. For instance, even though our VL-LB model retained the fundamental characteristics critical for viral function such as lipid domain formation, membrane curvature, and protein–lipid interactions, it did not include the full diversity of lipid species present in native HIV membranes. Thus, future studies could incorporate additional lipid species or explore larger-scale simulations to capture the effects of more complex lipid heterogeneity. In the same sense, HIV Env glycosylation plays a crucial role in shielding key epitopes and modulating antibody accessibility.^52^ While MPER-targeting antibodies like 10E8 are generally less affected by glycan variability compared to V1/V2 or V3-directed antibodies, glycosylation could still influence membrane organization and the local environment of MPER exposure.^40^ Therefore, future investigations may contemplate potential effects of glycosylation on the virus surface, and more generally, of the complex environment provided by immune cells, serum proteins, and additional host factors that may modulate 10E8 binding to membranes and neutralization mechanisms in vivo. Furthermore, we have selected 10E8 as the sole object of our study based on its unique membrane-accommodation traits, which may contribute to its exceptional breadth and potency, and on its consideration as a suitable focus for immunotherapy development, distinguishing it from other MPER-targeting antibodies. Despite these facts, neutralization-related membrane accommodation mechanisms should be generalized in the future to other classes of MPER bnAbs, such as 4E10- or LN01-like antibodies.

## Conclusions

Establishing the traits of Fab-membrane interactions underlying effective HIV-1 neutralization is key for developing new biologics and vaccines targeting MPER. Our comparative study reveals that changes in conformation and deeper insertion of the CDRH3 and FRL3 loops correlate with 10E8-mediated neutralization. Moreover, we detected function-related changes in the set of lipid interactions established by residues within those elements and within the CDRL1. Notably, the phospholipid binding site configured by chains and residues of FRL3, CDRL1, and CDRH3 was occupied almost exclusively by SPM in Fab/ctMPER-TMD complexes and inserted more deeply into the VL-LB in the Fab-Fus4/ctMPER-TMD system, which reproduced interactions of the most potent antibody. In conjunction, these observations lead to the conclusion that both ctMPER-TMD binding through the CDRH3 and deeper insertion into the membrane of the FRL3 loop are required for acquisition by 10E8 of a function-related phospholipid-binding site configuration. These findings warrant future 10E8 modifications combining unexplored sites of the FRL3 loop with newly developed compounds that may help sink more effectively the SPM-binding site into the membrane interface. In addition, when targeting the pan-neutralizing ctMPER epitope, liposome-based MPER vaccine formulations may require for their effectiveness the use of lipid mixtures that provide the appropriate nanoenvironment for SPM headgroup exposure and Fab scaffold insertion.

## Supporting Information Available

Neutralization curves; engagement of Fab 10E8 with ctMPER epitope in the simulations; lipids—CDRL1, FRL3, and CDRH3 contacts; contacts of lipids with selected residues of the CDRL1, FRL3, and CDRH3 segments; position of the phospholipid-binding site with respect to the bilayer plane in structures extracted from the densest clusters; illustration of the lipid nanoenvironment surrounding the SPM ligand; and somatically mutated residues in membrane-accommodating surfaces (PDF)

## Abbreviations

bnAb: broadly neutralizing antibody
Chol: cholesterol
CDRH3: complementarity-determining region 3 of the heavy chain
CDRL1: complementarity-determining region 1 of the light chain
FRL3: framework region 3 of the light chain
HC: heavy chain
LC: light chain
MPER: membrane-proximal external region
POPC: 1-palmitoyl-2-oleyl-*sn*-glycero-3-phosphocholine
POPE: 1-palmitoyl-2-oleoyl-*sn*-glycero-3-phosphoethanolamine
POPS: 1-palmitoyl-2-oleoyl-*sn*-glycero-3-phospho-*l*-serine
SPM: sphingomyelin
TMD: transmembrane domain
VL-LB: virus-like lipid bilayer
