# Supplementary material for: Distinctive Membrane Accommodation Traits Underpinning the Neutralization Activity of HIV-1 Antibody against MPER
Source: Mol Pharm. 2025 Apr 9;22(5):2494–508. doi: 10.1021/acs.molpharmaceut.4c01341 (PMC12056697; doi:10.1021/acs.molpharmaceut.4c01341)
Supplement: Supplementary file 1 — mp4c01341_si_001.pdf [file mp4c01341_si_001.pdf]

Supporting Information

Distinctive membrane accommodation traits underpinning neutralization activity of HIV-1 antibody against MPER

Carmen Domene[1], Brian Wiley[1,2], Sara Insausti[3,4], Edurne Rujas[3,5,6], and José L. Nieva[3,4*]*

[1]Department of Chemistry, University of Bath, Claverton Down, Bath, BA2 7AX, United Kingdom.
[2]ART-AI, Department of Computer Science, University of Bath, Claverton Down, Bath, BA2 7PB, United Kingdom
[3]Instituto Biofisika (CSIC, UPV/EHU), University of the Basque Country (UPV/EHU), P.O. Box 644, 48080 Bilbao, Spain
[4]Department of Biochemistry and Molecular Biology, University of the Basque Country (UPV/EHU) P.O. Box 644, 48080 Bilbao, Spain
[5]Department of Pharmacy and Food Sciences, Faculty of Pharmacy, University of the Basque Country (UPV/EHU), 01006 Vitoria, Spain
[6]Ikerbasque, Basque Foundation for Science, 48013 Bilbao, Spain

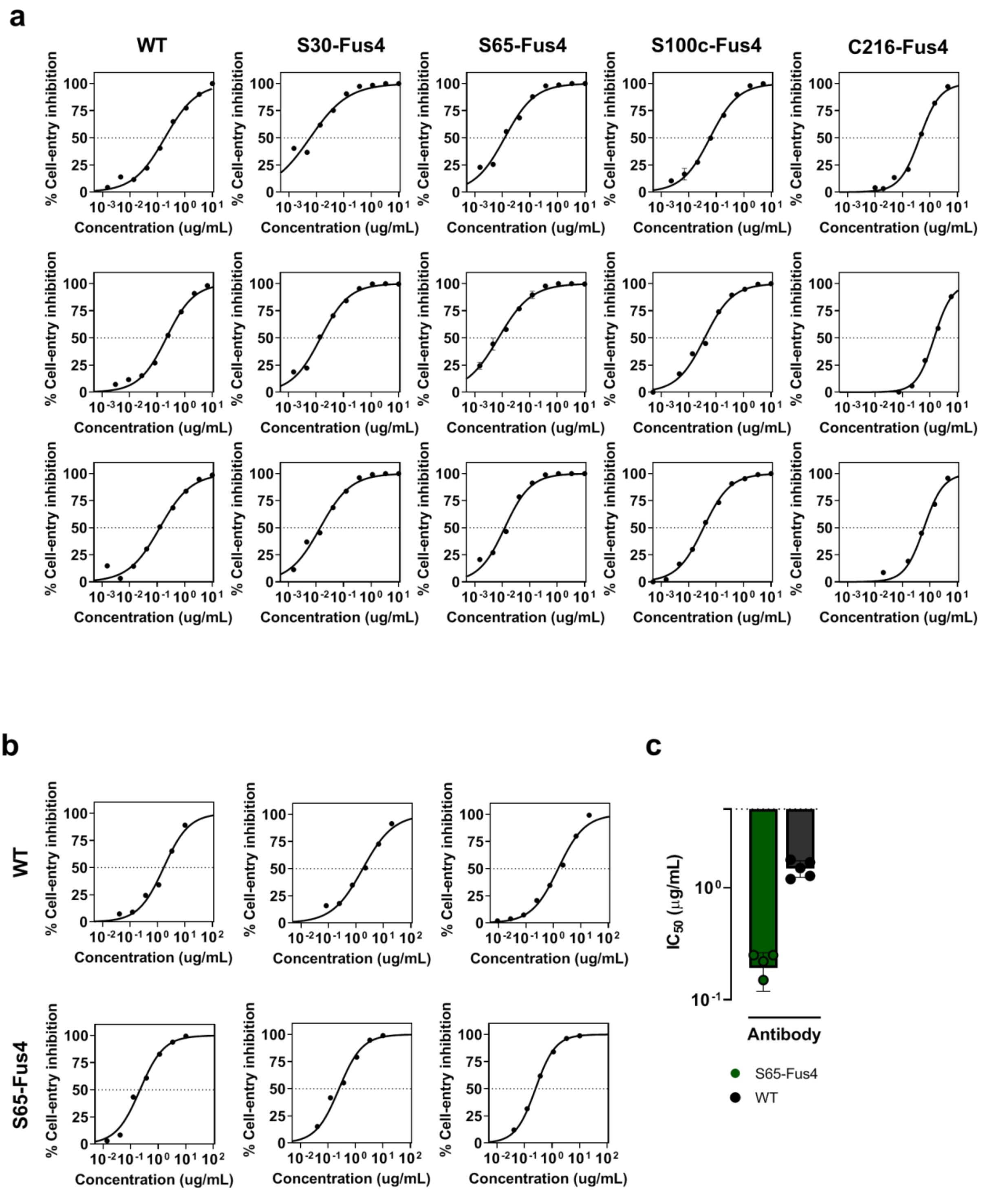

Supporting Figure 1. Dose-response curves of neutralization assays (a) Neutralization curves corresponding to data displayed in Figure 1 of the main manuscript. Points represent mean values from two replicate wells in a representative experiment. (b) Neutralization of Tier-3 PVO4 PsVs by the parental Fab 10E8 and chemically modified Fab-Fus4 (top and bottom panels respectively. (c) IC_{50} values inferred from the curves in the previous panel.

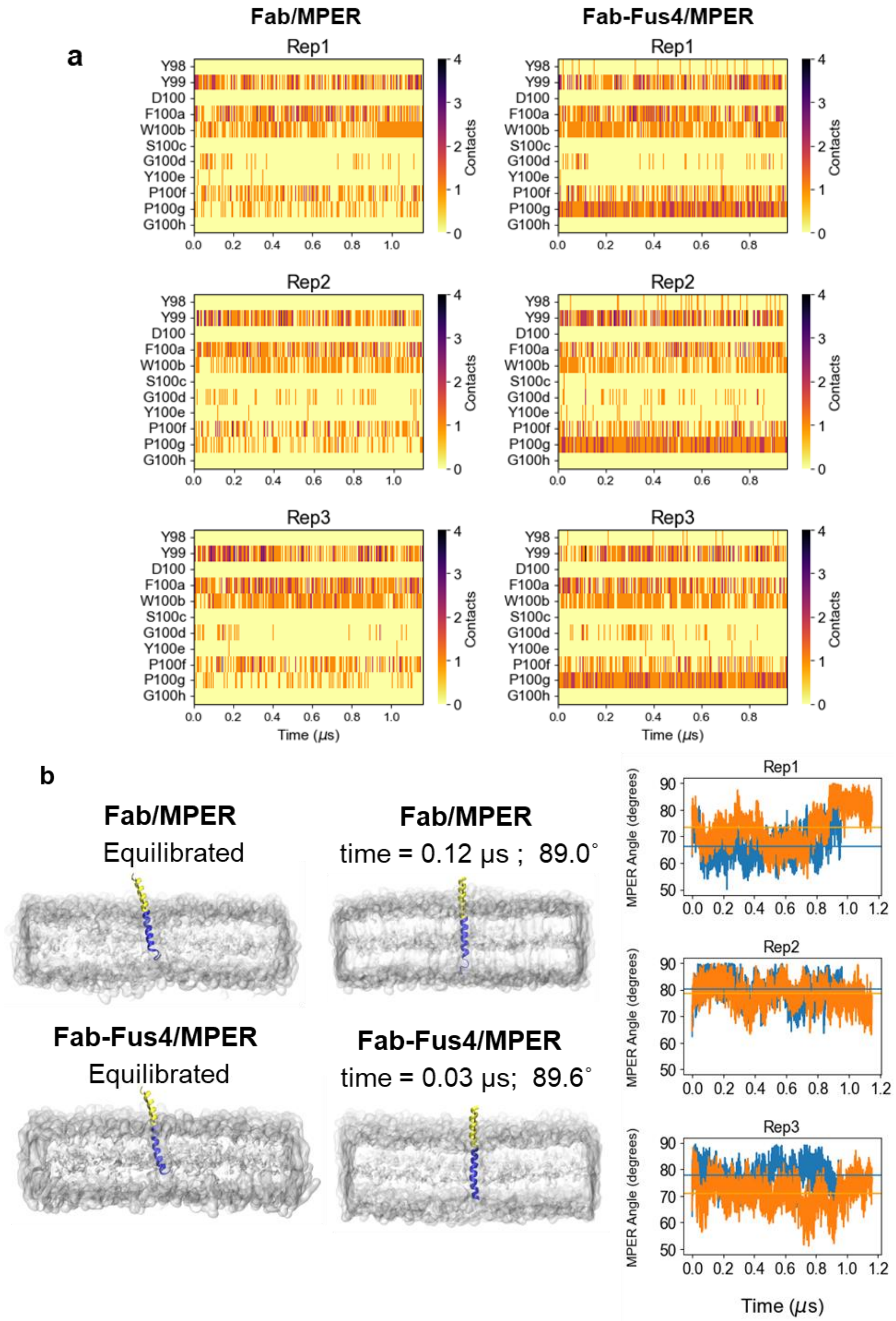

Supporting Figure 2. Engagement of Fab 10E8 with ctMPER epitope in the simulations. (a) Specific Fab-MPER helix contacts in 'Fab/MPER' and 'Fab-Fus4/MPER' systems; proportion of simulation time during which Fab residues maintain contacts with MPER residues. Contacts are defined as any heavy atom of antibody residues (paratope/Fab) being within 3.5 Å of any heavy atom in MPER (epitope). **(b)** Angle between ctMPER helix main axis and the plane at the average Z position of the phosphate groups in the upper leaflet.

Fab

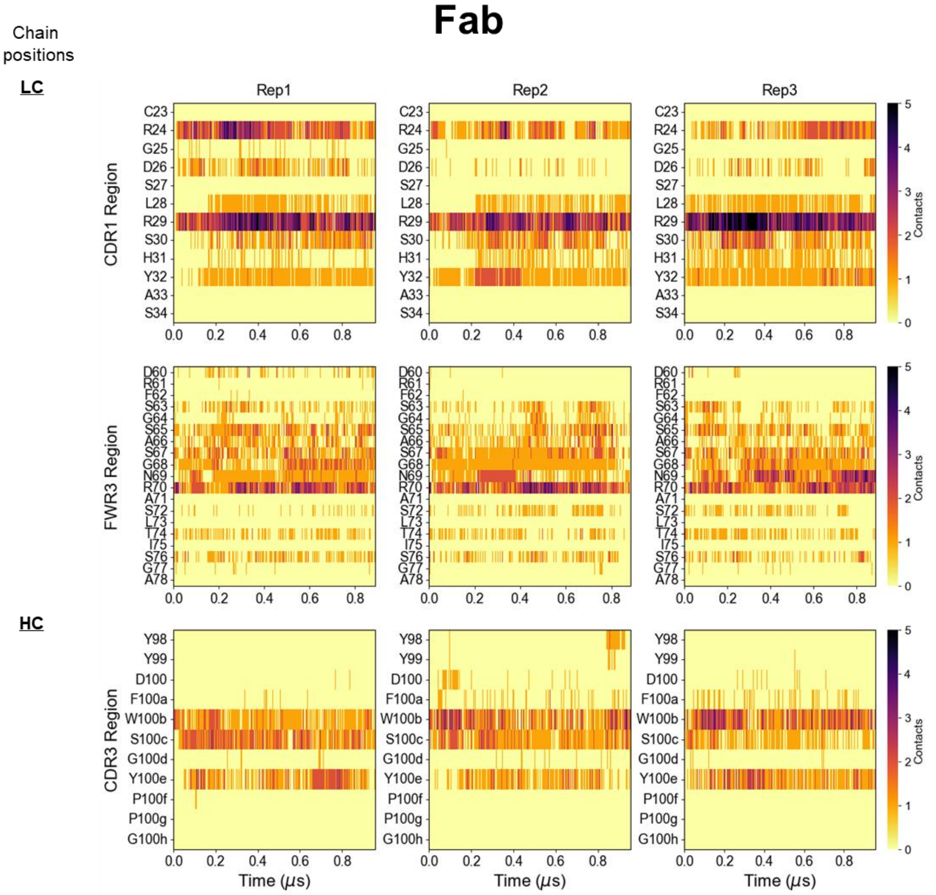

Chain positions
LC
HC
Rep1
Rep2
Rep3
CDR1 Region
FWR3 Region
CDR3 Region
C23 R24 G25 D26 S27 L28 R29 S30 H31 Y32 A33 S34
D60 R61 F62 S63 G64 S65 A66 S67 G68 N69 R70 A71 S72 L73 T74 I75 S76 G77 A78
Y98 Y99 D100 F100a W100b S100c G100d Y100e P100f P100g G100h
Contacts
Time (μs)

Fab/MPER

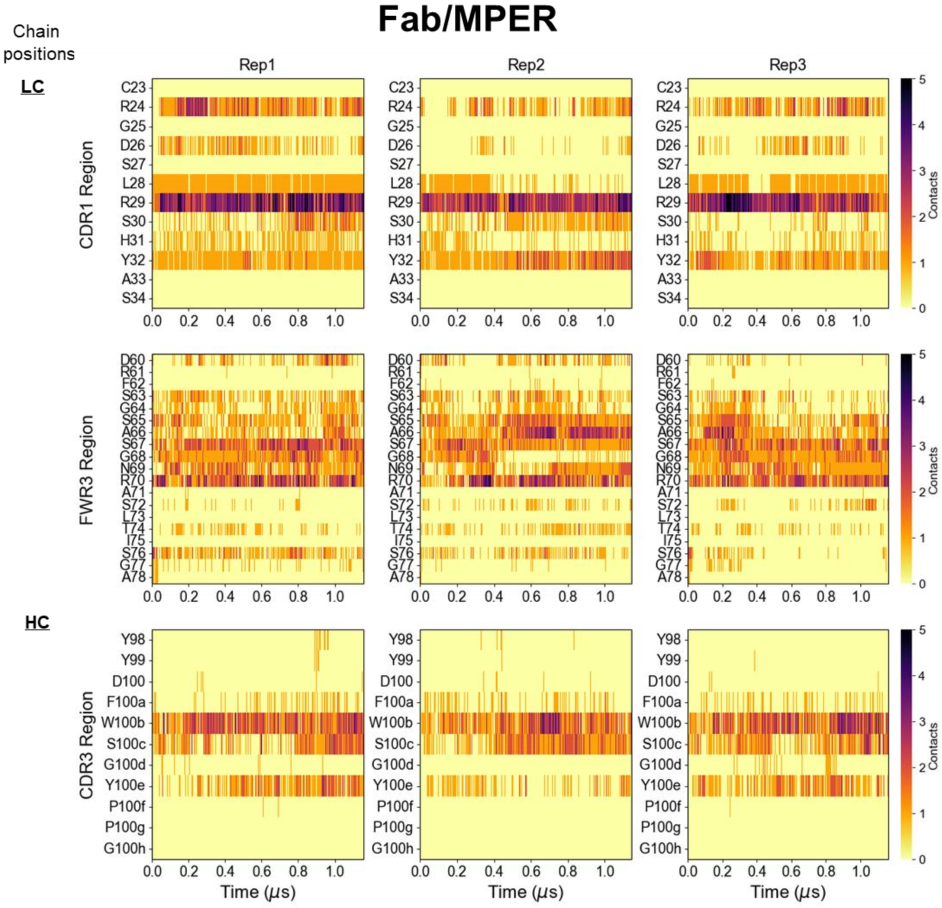

Chain positions
LC
HC
Rep1
Rep2
Rep3
CDR1 Region
FWR3 Region
CDR3 Region
C23 R24 G25 D26 S27 L28 R29 S30 H31 Y32 A33 S34
D60 R61 F62 S63 G64 S65 A66 S67 G68 N69 R70 A71 S72 L73 T74 I75 S76 G77 A78
Y98 Y99 D100 F100a W100b S100c G100d Y100e P100f P100g G100h
Contacts
Time (μs)

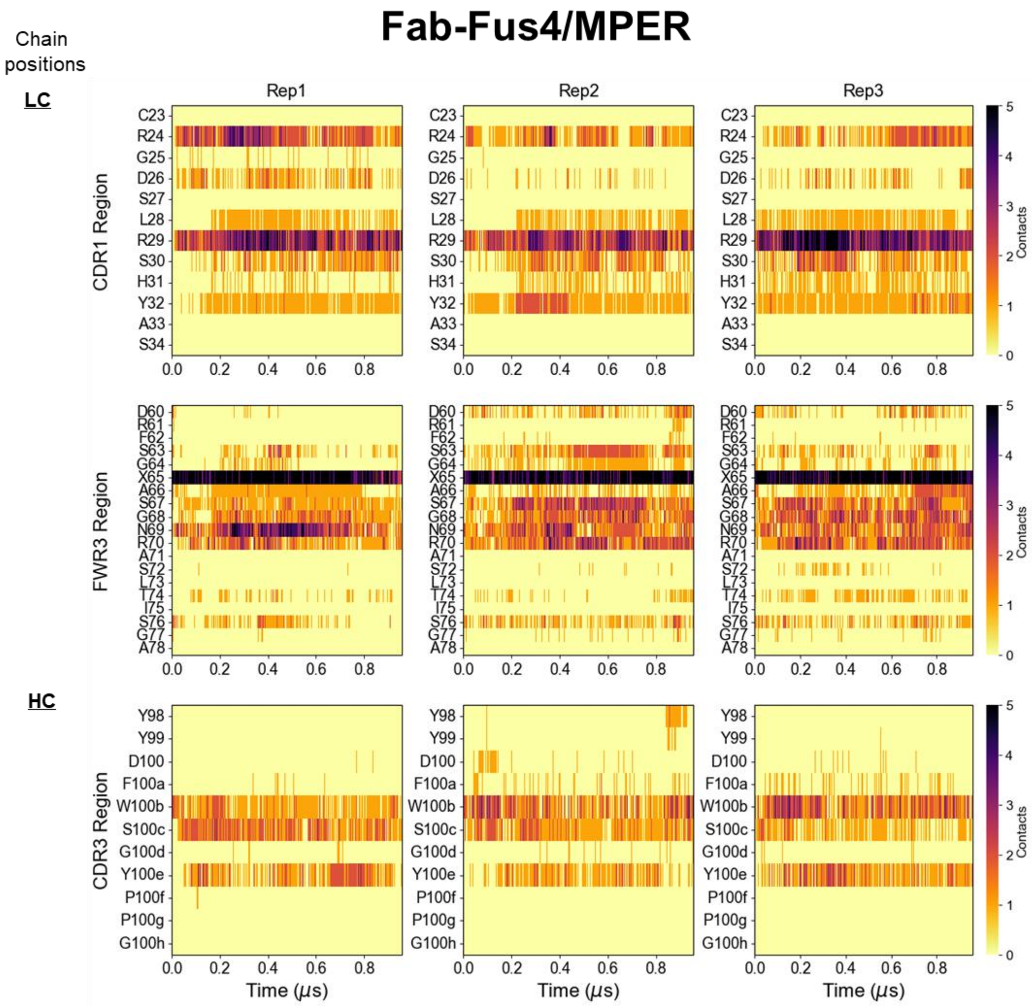

Supporting Figure 3. CDRL1, FRL3 and CDRH3 residues in contact with lipids during the simulations. Contacts between any lipid are summed to a total number of lipid residues in contact with the loop residues. Contacts were defined as instances where any heavy atom of a lipid residue approached within 3.5 Å of the heavy atoms of each individual protein residue.

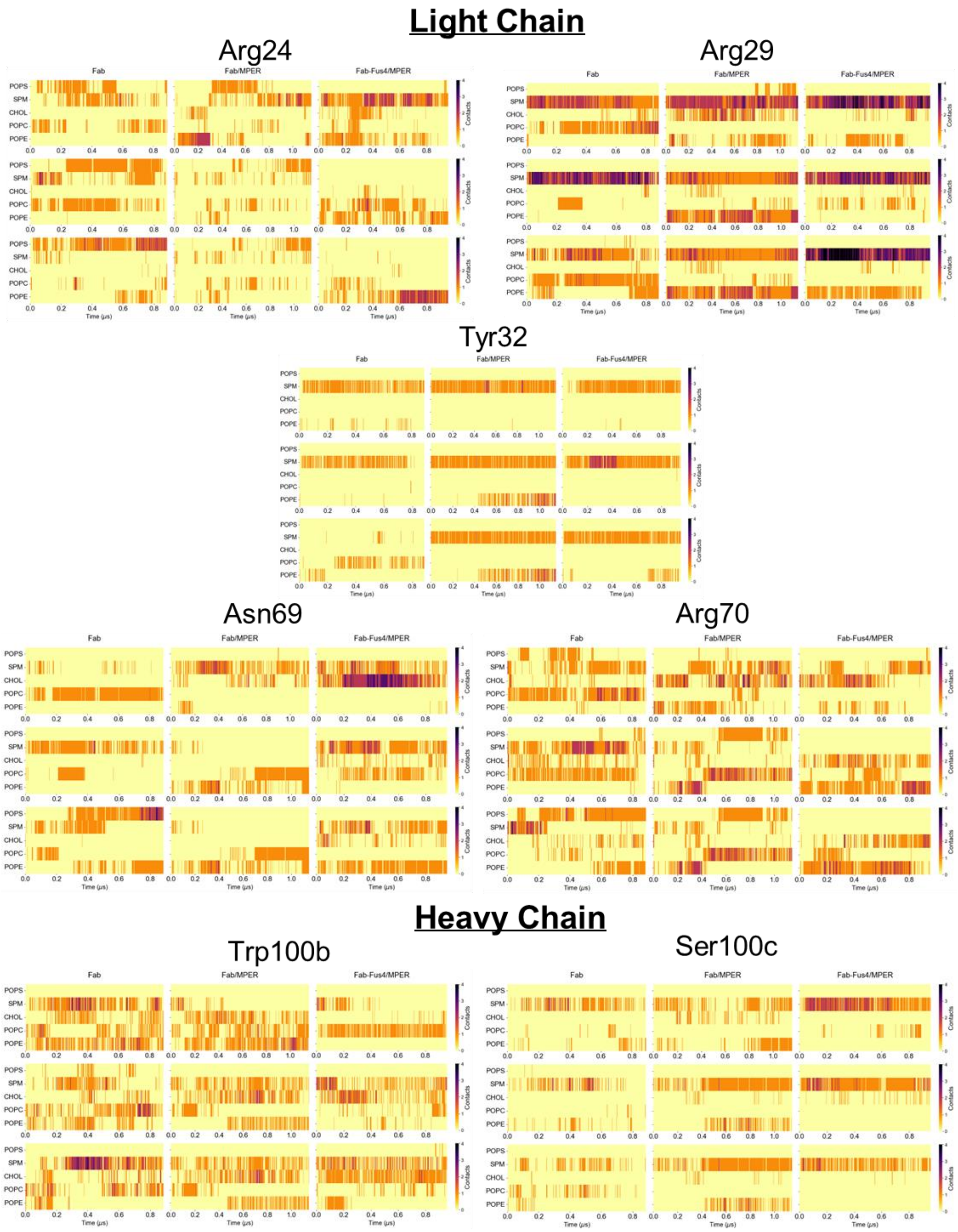

Supporting Figure 4. Lipid interactions were assessed with selected residues of the CDRL1, FRL3, and CDRH3 segments. Contacts were defined as instances where any heavy atom of a lipid residue approached within 3.5 Å of the heavy atoms of each individual protein residue.

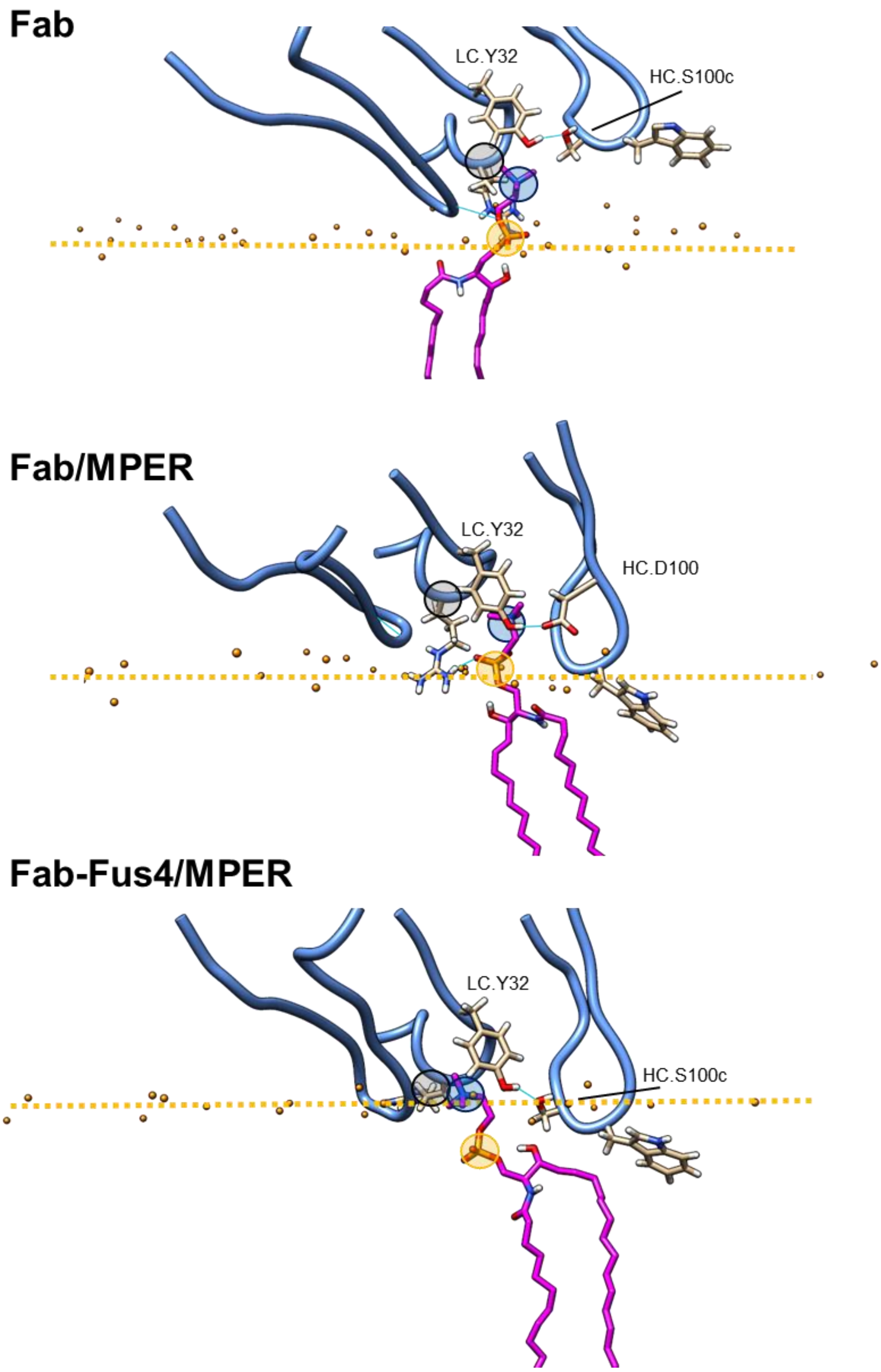

Supporting Figure 5. Position of the phospholipid-binding site with respect to the bilayer plane in structures extracted from the densest clusters. Displayed views are rotated 180º with respect to those shown in Figure 5b of the main text.

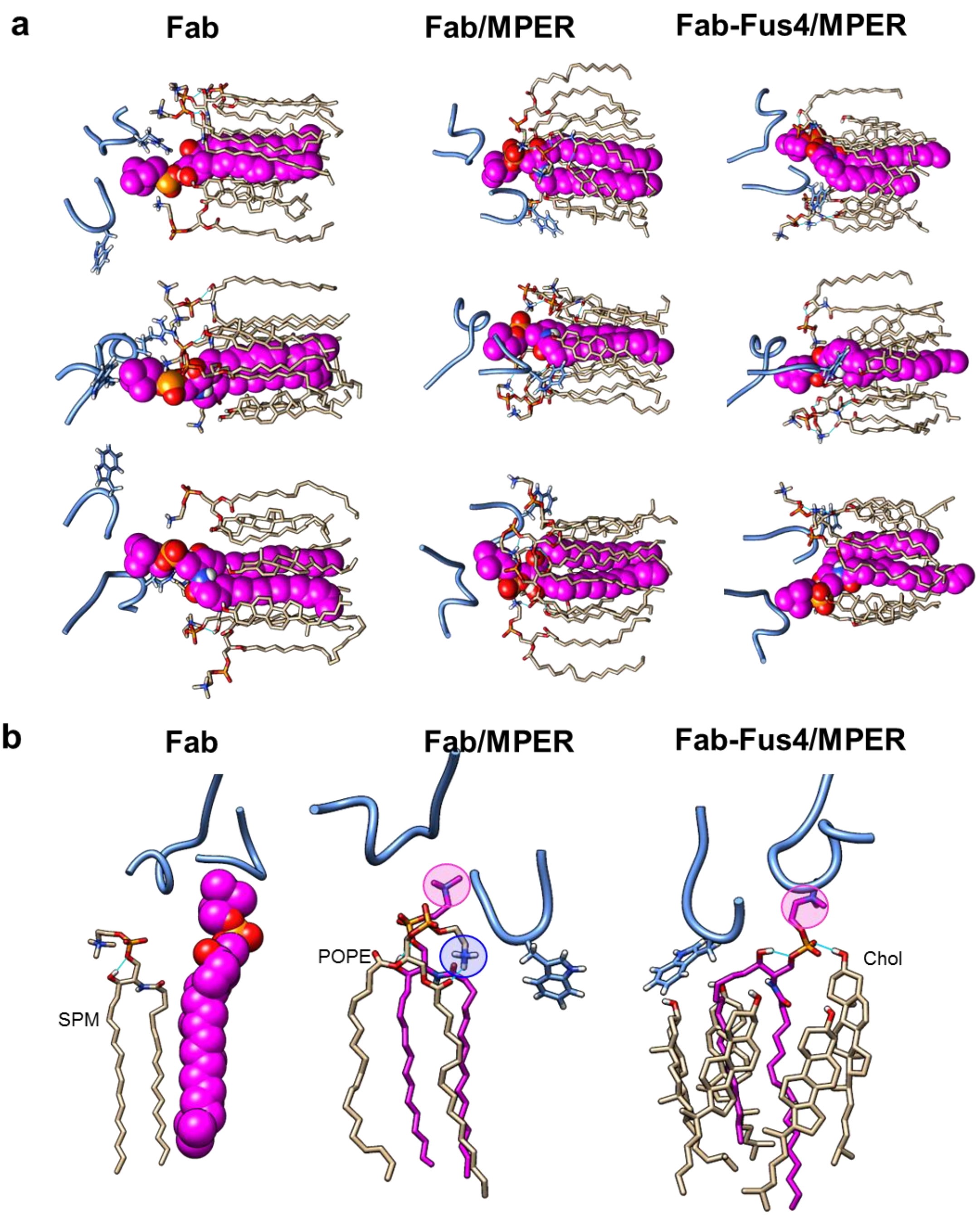

Supporting Figure 6. Lipid nano-environment surrounding the SPM ligand. (a) VL-LB lipids making contact (distance < 3.5 Å) with Fab-bound SPM molecule (VW surface in magenta). **(b)** Different interaction motifs sustaining exposure of SPM ligand's phosphocholine moiety (see main text).

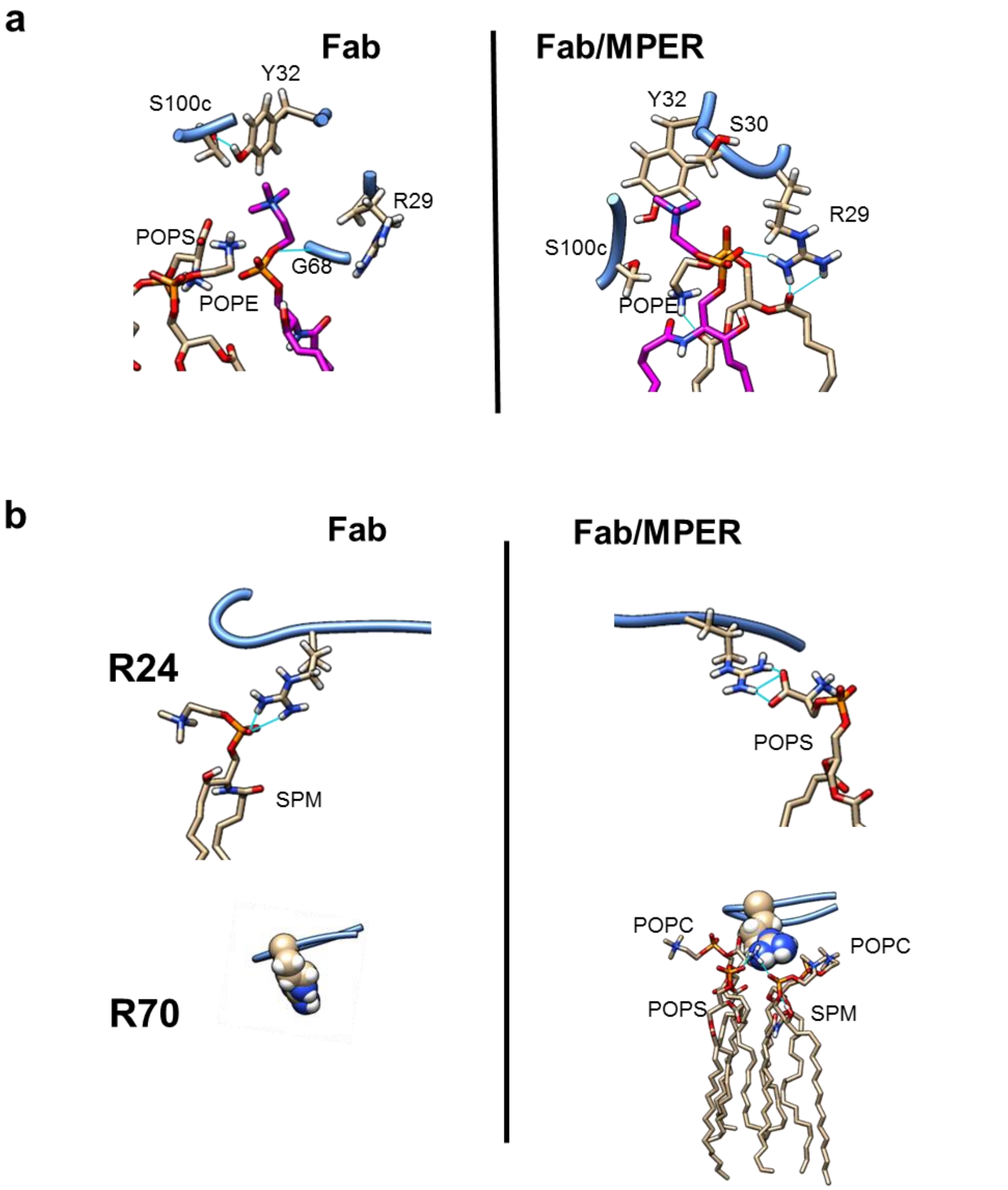

Supporting Figure 7. Somatically mutated residues in membrane-accommodating surfaces. (a) SPM-binding sites of the 'Fab' and 'Fab/MPER' structures derived from the cluster analyses. Side chains of displayed residues established contacts with the phosphocholine moiety at distances <3.5 Å. **(b)** Interactions with VL-LB lipids (distance<3.5 Å) of LC.Arg24 and LC.Arg70 in the same structures.
